# Supplementary material for: Inhibition of proteasome rescues a pathogenic variant of respiratory chain assembly factor COA7
Source: EMBO Mol Med. 2019 Mar 18;11(5):e9561. doi: 10.15252/emmm.201809561 (PMC6505684; doi:10.15252/emmm.201809561)
Supplement: Supplementary file 1 — Appendix [file EMMM-11-e9561-s001.pdf]

Appendix

Inhibition of proteasome rescues a pathogenic variant of respiratory chain assembly factor COA7

Karthik Mohanraj, Michal Wasilewski, Cristiane Benincá, Dominik Cysewski, Jarosław Poznanski, Paulina Sakowska, Zaneta Bugajska, Markus Deckers, Sven Dennerlein, Erika Fernandez-Vizarra, Peter Rehling, Michal Dadlez, Massimo Zeviani, Agnieszka Chacinska

Table of Contents

Appendix Figure S1.. 3

Appendix Figure S2.. 4

Appendix Figure S3.. 5

Appendix Figure S4.. 6

Appendix Figure S5.. 7

A

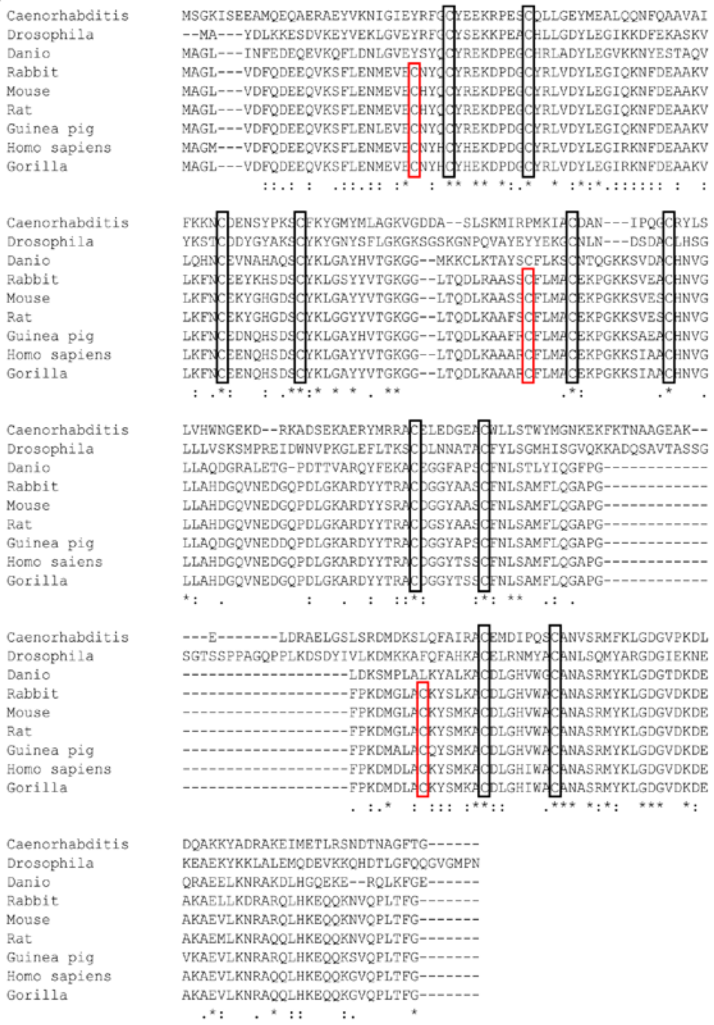
Caenorhabditis MSGKISEEAMQEQAERAEYVKNIGIEYRFGCYEEKRPESCQLLGEYMEALQQNFQAAVAI
Drosophila --MA---YDLKKESDVKEYVEKLGVEYRFGCYSEKKPEACHLLGDYLEGIKKDFEKASKV
Danio MAGL---INFEDEQEVKQFLDNLGVEYSYQCYREKDPEGCHRLADYLEGVKKNYESTAQV
Rabbit MAGL---VDFQDEEQVKSFLENMEVECNYQCYREKDPDGCYRLVDYLEGIQKNFDEAAKV
Mouse MAGL---VDFQDEEQVKSFLENMEVECHYQCYREKDPEGCYRLVDYLEGIQKNFDEAAKV
Rat MAGL---VDFQDEEQVKSFLENMEVECHYQCYREKDPEGCYRLVDYLEGIQKNFDEAAKV
Guinea pig MAGL---VDFQDEEQVKSFLENLEVECNYQCYREKDPDGCYRLVDYLEGIQKNFDEAAKV
Homo sapiens MAGM---VDFQDEEQVKSFLENMEVECNYHCYHEKDPDGCYRLVDYLEGIRKNFDEAAKV
Gorilla MAGL---VDFQDEEQVKSFLENMEVECNYHCYHEKDPDGCYRLVDYLEGIRKNFDEAAKV
Caenorhabditis FKKNCDENSYPKSCFKYGMYMLAGKVGDDA--SLSKMIRPMKIACDAN---IPQGCRYLS
Drosophila YKSTCDDYGYAKSCYKYGNYSFLGKGKSGSKGNPQVAYEYYEKGCNLN---DSDACLHSG
Danio LQHNCEVNAHAQSCYKLGAYHVTGKGG--MKKCLKTAYSCFLKSCNTQGKKSVDACHNVG
Rabbit LKFNCEEYKHSDSCYKLGSYYVTGKGG--LTQDLRAASSCFLMACEKPGKKSVEACHNVG
Mouse LKFNCEKYGHGDSCYKLGAYYVTGKGG--LTQDLKAASSCFLMACEKPGKKSVESCHNVG
Rat LKFNCEKYGHGDSCYKLGGYYVTGKGG--LTQDLKAAFSCFLMACEKPGKKSVESCHNVG
Guinea pig LKFNCEDNQHSDSCYKLGAYHVTGKGG--LTQDLKAAFRCFLMACEKPGKKSAEACHNVG
Homo sapiens LKFNCEENQHSDSCYKLGAYYVTGKGG--LTQDLKAAARCFLMACEKPGKKSIAACHNVG
Gorilla LKFNCEENQHSDSCYKLGAYYVTGKGG--LTQDLKAAARCFLMACEKPGKKSIAACHNVG
Caenorhabditis LVHWNGEKD--RKADSEKAERYMRRACELEDGEACWLLSTWYMGNKEKFKTNAAGEAK--
Drosophila LLLVSKSMPREIDWNVPKGLEFLTKSCDLNNATACFYLSGMHISGVQKKADQSAVTASSG
Danio LLAQDGRALETG-PDTTVARQYFEKACEGGFAPSCFNLSTLYIQGFPG------------
Rabbit LLAHDGQVNEDGQPDLGKARDYYTRACDGGYAASCFNLSAMFLQGAPG------------
Mouse LLAHDGQVNEDGQPDLGKARDYYSRACDGGYAASCFNLSAMFLQGAPG------------
Rat LLAHDGQVNEDGQPDLGKARDYYTRACDGSYAASCFNLSAMFLQGAPG------------
Guinea pig LLAQDGQVNEDDQPDLGKARDYYTRACDGGYAPSCFNLSAMFLQGAPG------------
Homo saiens LLAHDGQVNEDGQPDLGKARDYYTRACDGGYTSSCFNLSAMFLQGAPG------------
Gorilla LLAHDGQVNEDGQPDLGKARDYYTRACDGGYTSSCFNLSAMFLQGAPG------------
Caenorhabditis ---E-------LDRAELGSLSRDMDKSLQFAIRACEMDIPQSCANVSRMFKLGDGVPKDL
Drosophila SGTSSPPAGQPPLKDSDYIVLKDMKKAFQFAHKACELRNMYACANLSQMYARGDGIEKNE
Danio -------------------LDKSMPLALKYALKACDLGHVWGCANASRMYKLGDGTDKDE
Rabbit -------------------FPKDMGLACKYSLKACDLGHVWACANASRMYKLGDGVDKDE
Mouse -------------------FPKDMGLACKYSMKACDLGHVWACANASRMYKLGDGVDKDE
Rat -------------------FPKDMGLACKYSMKACDLGHVWACANASRMYKLGDGVDKDE
Guinea pig -------------------FPKDMALACQYSMKACDLGHVWACANASRMYKLGDGVDKDE
Homo sapiens -------------------FPKDMDLACKYSMKACDLGHIWACANASRMYKLGDGVDKDE
Gorilla -------------------FPKDMDLACKYSMKACDLGHIWACANASRMYKLGDGVDKDE
Caenorhabditis DQAKKYADRAKEIMETLRSNDTNAGFTG------
Drosophila KEAEKYKKLALEMQDEVKKQHDTLGFQQGVGMPN
Danio QRAEELKNRAKDLHGQEKE--RQLKFGE------
Rabbit AKAELLKDRARQLHKEQQKNVQPLTFG-------
Mouse AKAEVLKNRARQLHKEQQKNVQPLTFG-------
Rat AKAEMLKNRAQQLHKEQQKNVQPLTFG-------
Guinea pig VKAEVLKNRARQLHKEQQKSVQPLTFG-------
Homo sapiens AKAEVLKNRAQQLHKEQQKGVQPLTFG-------
Gorilla AKAEVLKNRAQQLHKEQQKGVQPLTFG-------

B

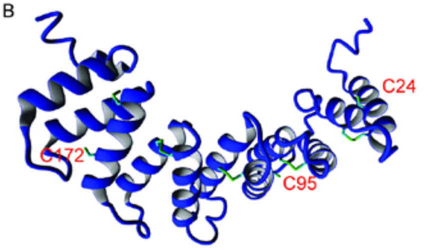
C24
C172
C95

C

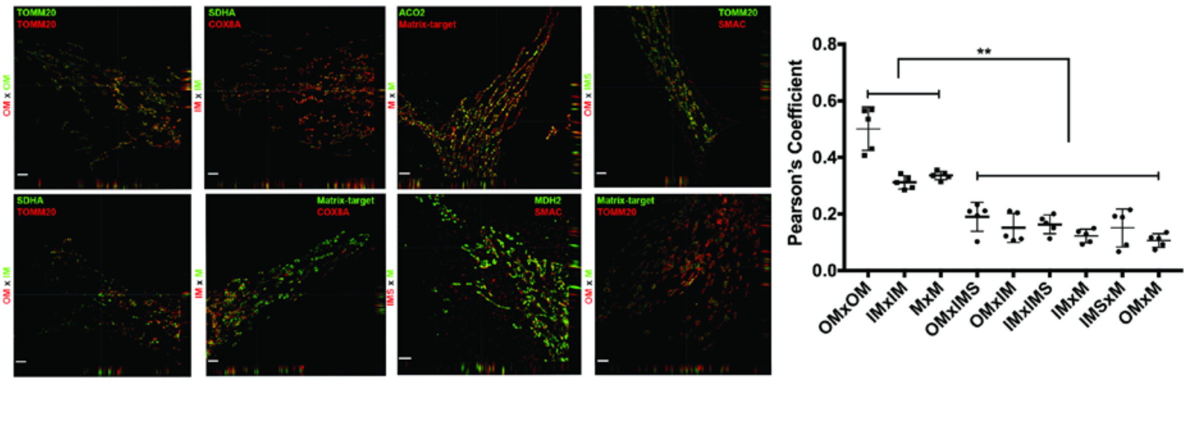
TOMM20
TOMM20
SDHA
COX8A
ACO2
Matrix-target
TOMM20
SMAC
SDHA
TOMM20
Matrix-target
COX8A
MDH2
SMAC
Matrix-target
TOMM20
Pearson's Coefficient
0.8
0.6
0.4
0.2
0.0
**
OMxOM
IMxIM
MxM
OMxIMS
OMxIM
IMxIMS
IMxM
IMSxM
OMxM

Appendix Figure S1. Evolutionary conservation of cysteine residues and homology modeling of COA7 (Corresponding to Figure 2).

(A) Multiple sequence alignment of COA7 sequence across metazoans. Red boxes indicate cysteine residues that are present only in mammals. Black boxes indicate cysteine residues that are well conserved across eukaryotes.
(B) Homology-modeled structure of COA7 that depicts cysteine residues that are involved in disulfide bonds based on homology modeling.
(C) N-SIM super-resolution micrographs of one Z-stack (0.15 μm) orthogonal section (XYZ) of Hela cells transfected with different subcompartment markers TOMM20-DsRed (OM), COX8A-DsRed (IM), and mtPAGFP (Matrix-target) labeled with anti-TOMM20 (OM), anti-SDHA (IM), anti-Aconitase 2 (M) and Smac/Diablo (IMS) antibodies. The picture represents the majority population of cells from three independent experiments. Scale bar = 2 μm. The panel shows Pearson's coefficient in a co-localized volume of different subcompartment combinations. The data are expressed as a mean ± SD (n=5). **p<0.01 [OMxOM vs. OMxIMS p<0.0001; OMxOM vs. OMxIM p<0.0001; OMxOM vs. IMxIMS p<0.0001; OMxOM vs. IMxM p<0.0001; OMxOM vs. IMSxM p<0.0001; OMxOM vs. OMxM p<0.0001; IMxIM vs. OMxIMS p=0.0043; IMxIM vs. OMxIM p<0.0001; IMxIM vs. IMxIMS p=0.0003; IMxIM vs. IMxM p<0.0001; IMxIM vs. IMSxM p<0.0001, IMxIM vs. OMxM p<0.0001; MxM vs. OMxIMS p=0.0004; MxM vs. OMxIM p=0.0001; MxM vs. IMxIMS p=0.0001; MxM vs. IMxM p=0.0001; MxM vs. IMSxM p=0.0001; MxM vs. OMxM p=0.0001] (one-way ANOVA). IM – inner membrane, IMS – intermembrane space, M – matrix, OM – outer membrane

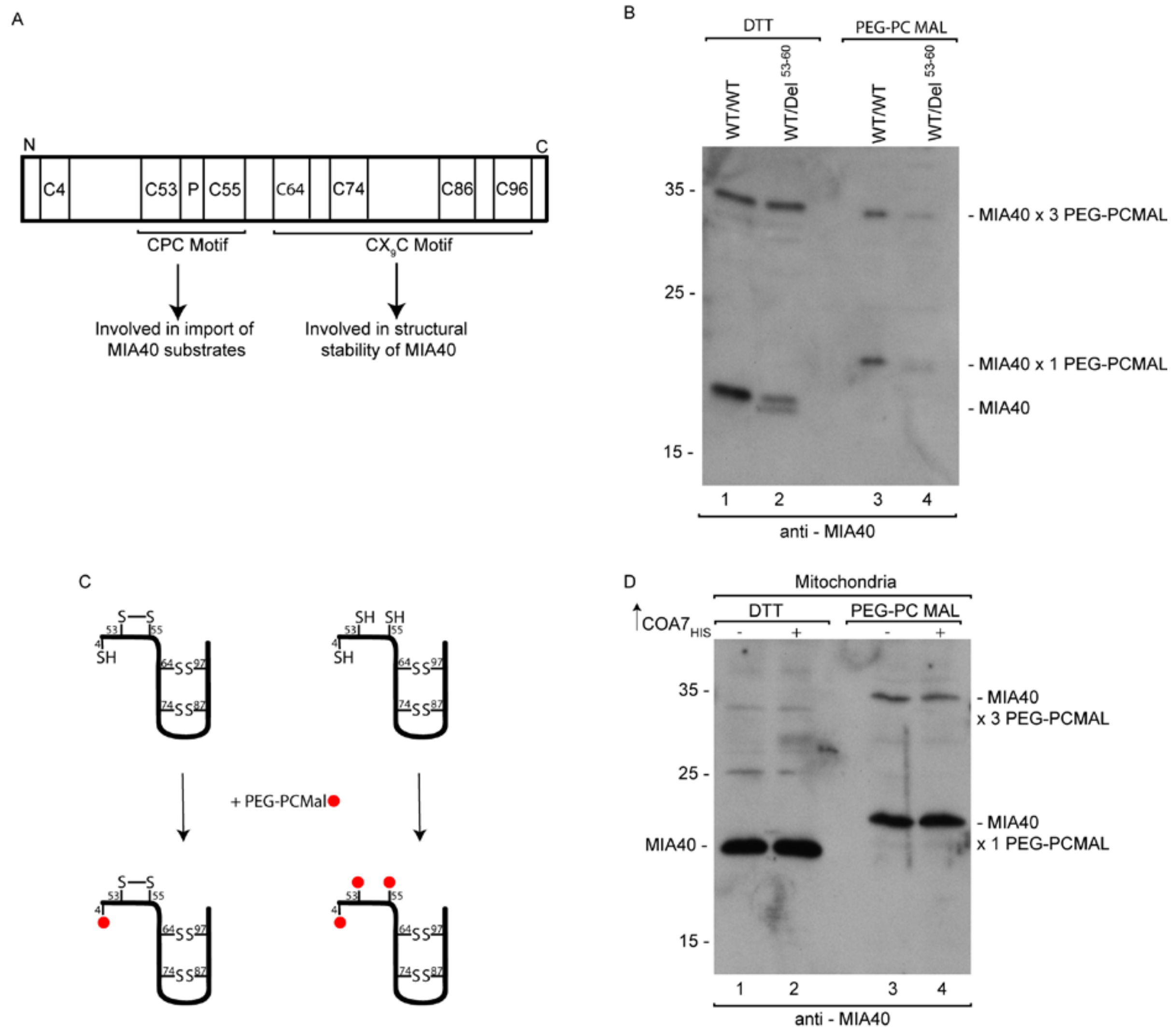

Appendix Figure S2. Redox state of MIA40 in COA7 overexpressing cells (Corresponding to Figure 3).

(A) Schematic representation of cysteine residues and its function in MIA40.
(B) Mitochondria were isolated from HEK293 WT/WT and HEK293 MIA40 WT/Del$^{53\text{-}60}$ cells. The mitochondria were then treated with either DTT or PEG-PCMal, a modifying agent that binds to free cysteine residues. The reagent provides a shift of 5 kDa upon binding to each cysteine residue. The samples were analyzed by SDS-PAGE and Western blot.
(C) Schematic representation of the thiol trapping assay using PEG –PCMal.
(D) Mitochondria isolated from HEK293 cells transiently overexpressing $COA7_{HIS}$ were treated with either DTT or PEG-PCMal. The samples were analyzed by SDS-PAGE and Western blot.

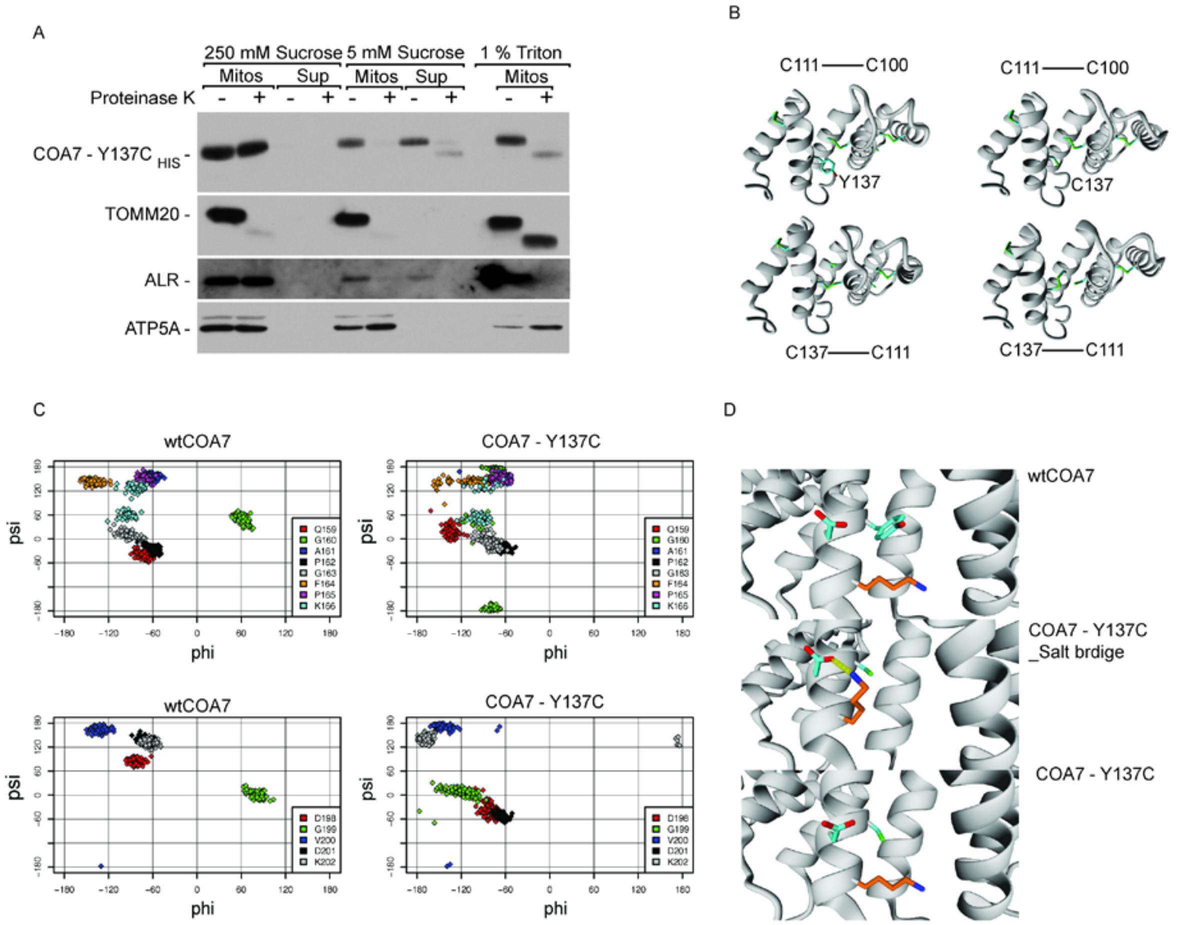

Appendix Figure S3. Sub-mitochondrial localization and molecular modeling of mutant COA7-Y137C (Corresponding to Figure 6).

(A) Localization of overexpressed COA7-Y137C analyzed by limited degradation by proteinase K in intact mitochondria (250 mM sucrose), mitoplasts (5 mM sucrose), and mitochondrial lysates (1 % Triton X-100) that were isolated from HEK293 cells. The samples were analyzed by SDS-PAGE and Western blot. Mitos, mitochondria; Sup, post-mitochondria supernatant.

(B) Homology-modeled structure that depicts alternate disulfide bonds in the COA7-Y137C mutant.

(C) Ramachandran plot of amino acids in the loop region of the COA7-Y137C mutant. Flexibility of the loop regions was quantified by backbone dihedral angles (phi and psi) of individual residues, variations of which were plotted in the Ramachandran plots. The conformation of G160 and G199 differed substantially upon Y137C replacement, and the conformation of Q159, D198, D201, and K202 was also altered. The analyzed residues had wider distributions of dihedral angles (phi and psi) in the COA7-Y137C mutant, indicating greater flexibility of these loops. No significant change was observed in the conformation of the other loops in the COA7-Y137C mutant. Wt, wild-type.

(D) Salt bridge formation in the COA7-Y137C mutant. Wildtype COA7 and mutant COA7 were subjected to molecular dynamics simulations, and a snapshot of salt bridge formation in the mutant is presented.

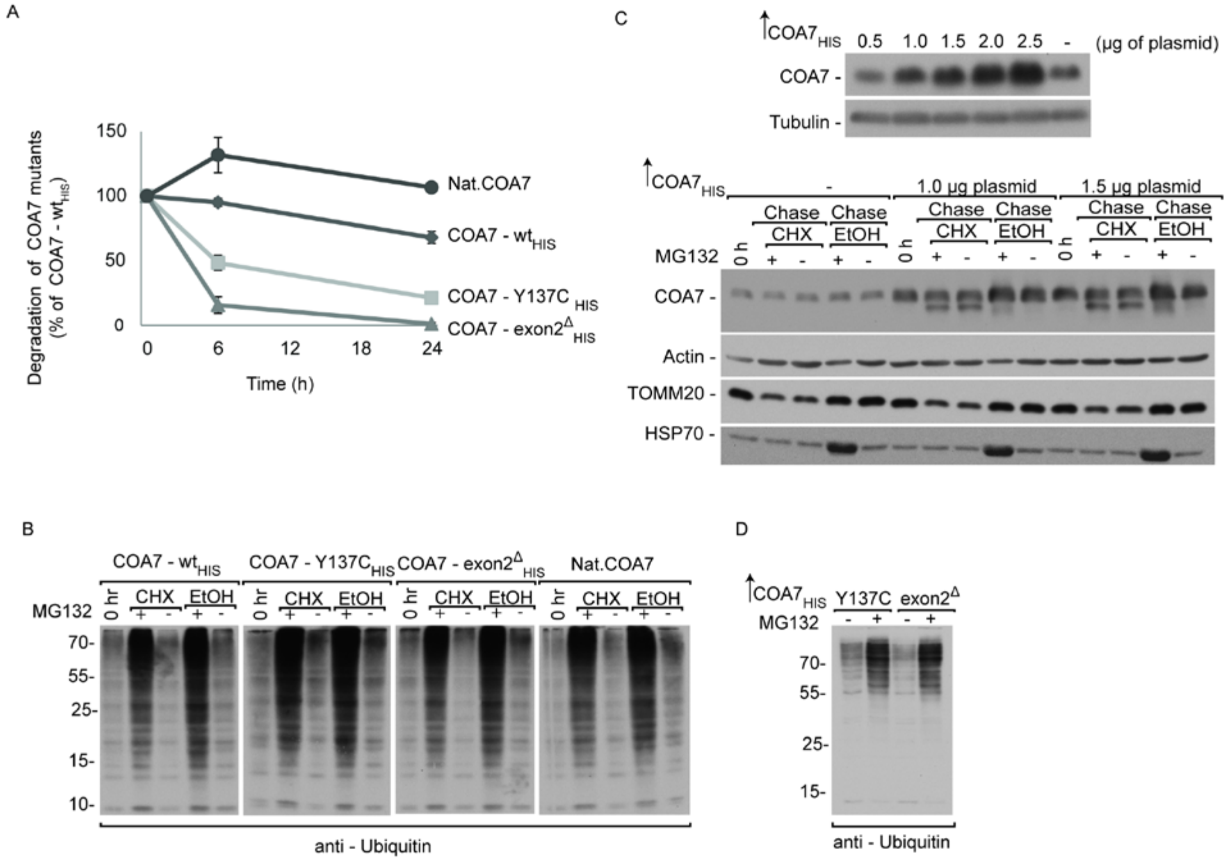

Appendix Figure S4. Degradation of COA7 mutants by proteasome (Corresponding to Figure 7).

(A) HEK293 cells that expressed wildtype or mutant $COA7_{HIS}$ were treated with CHX for the indicated times, and protein extracts were isolated. The samples were analyzed by reducing SDS-PAGE and Western blot. The results of three biological replicates were analyzed, quantified, and normalized to the level of native COA7 at time 0h. The data are expressed as a mean ± SEM (n=3). Nat, native.

(B) Cellular protein extracts of HEK293 cells transfected with different amounts of plasmid encoding $COA7_{HIS}$. HEK293 cells that expressed different amounts of wildtype $COA7_{HIS}$ were treated with CHX and/or MG132 for the indicated times, and protein extracts were isolated. The samples were analyzed by reducing SDS-PAGE and Western blot. CHX, cycloheximide.

(C) Samples from Fig 7D analyzed by SDS-PAGE and Western blot against ubiquitin.

(D) Samples from Fig 7F analyzed by SDS-PAGE and Western blot against ubiquitin.

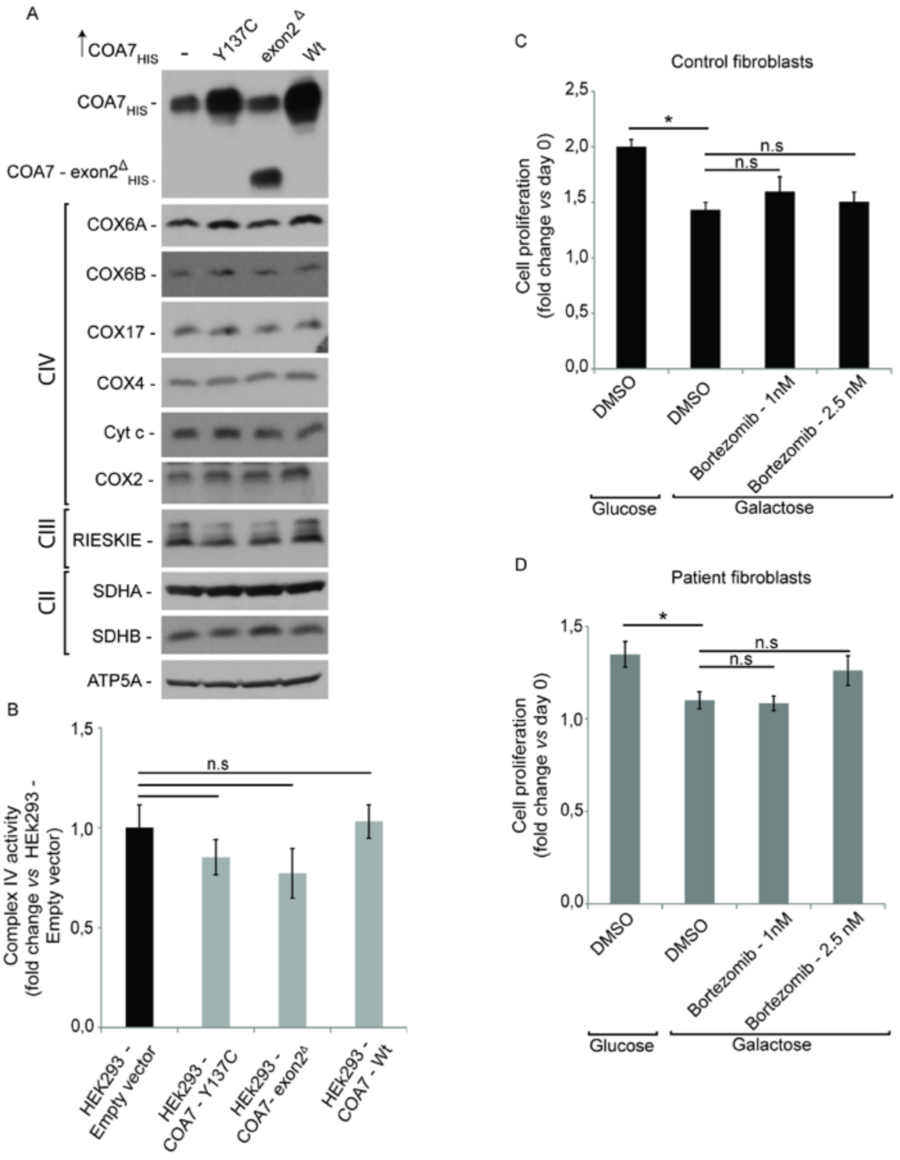

Appendix Figure S5. Effects of wildtype and mutant COA7 overexpression on complex IV. Cell proliferation during bortezomib treatment (corresponding to Figure 9).

(A) Cellular protein extracts from HEK293 cells that were transfected with different amount of plasmid encoding wildtype and mutants of $COA7_{HIS}$ were isolated. The samples were analyzed by reducing SDS-PAGE and Western blot.

(B) HEK293 cells were transfected with a plasmid that encoded wildtype or mutant COA7 (COA7-Y137C and COA7-exon2$^{\Delta}$) for 24h. The fibroblasts were harvested, and complex IV activity was measured in digitonized cellular extracts. Activity is expressed as millimoles of oxidized cytochrome *c* per minute per milligram of protein. The results of three biological replicates were analyzed, quantified, and normalized to empty vector-bortezomib treated samples and the data are expressed as a mean ± SEM (n=3).

(C) Control immortalized human fibroblasts (Ai) were grown in glucose or galactose DMEM for 24h in the presence of indicated concentrations of bortezomib. Cells were counted and cell proliferation was expressed as a fold change of cell count on day 1 *vs* day 0 ± SEM. (n=3; *p=0.003)

(D) Patient-derived immortalized human fibroblasts (mt4229i) were grown in glucose or galactose DMEM for 24h in the presence of indicated concentrations of bortezomib. Cells were counted and cell proliferation was expressed as a fold change of cell count on day 1 *vs* day 0 ± SEM. (n=4; *p=0.02)
