# Supplementary material for: Inhibition of proteasome rescues a pathogenic variant of respiratory chain assembly factor COA7
Source: EMBO Mol Med. 2019 Mar 18;11(5):e9561. doi: 10.15252/emmm.201809561 (PMC6505684; doi:10.15252/emmm.201809561)
Supplement: Supplementary file 2 — Expanded View Figures PDF [file EMMM-11-e9561-s002.pdf]

Expanded View Figures

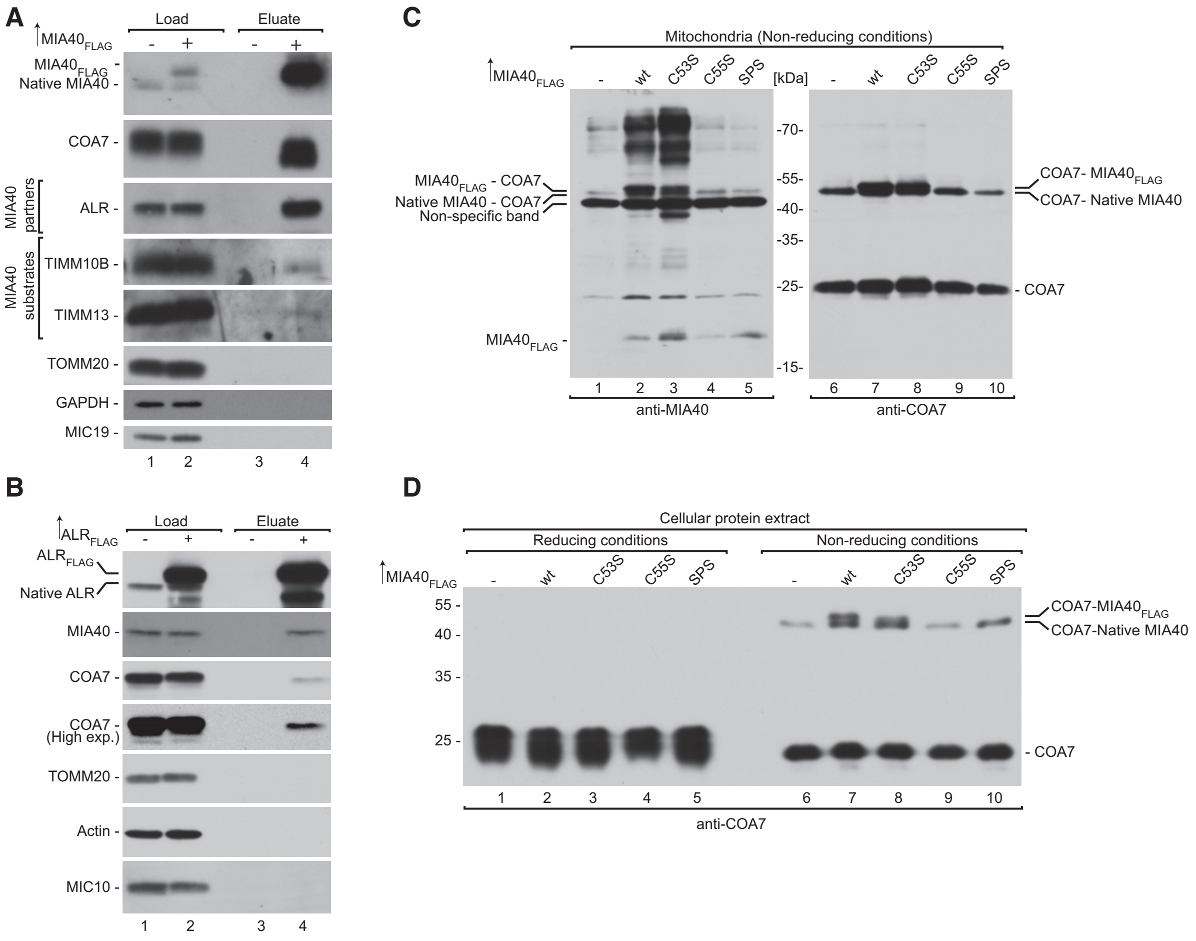

Figure EV1. COA7 interacts with MIA40 by disulfide bonding.

A Flp-In T-REx 293 cells induced to express $MIA40_{FLAG}$ were solubilized, and the affinity purification of $MIA40_{FLAG}$ was performed. The fractions were analyzed by SDS–PAGE and Western blot. Load: 2.5%. Eluate: 100%.

B Flp-In T-REx 293 cells induced to express ALR_{FLAG} were solubilized, and the affinity purification of ALR_{FLAG} was performed. The fractions were analyzed by SDS–PAGE and Western blot. Load: 2.5%. Eluate: 100%.

C Mitochondria were isolated from Flp-In T-REx 293 cells induced to express wild-type and mutant forms of $MIA40_{FLAG}$ under non-reducing conditions. The extract was analyzed by non-reducing SDS–PAGE and Western blot.

D Cellular protein extracts were isolated from Flp-In T-REx 293 cells induced to express wild-type and mutant forms of $MIA40_{FLAG}$ under non-reducing conditions. The extract was analyzed by non-reducing SDS–PAGE and Western blot.

© 2019 The Authors

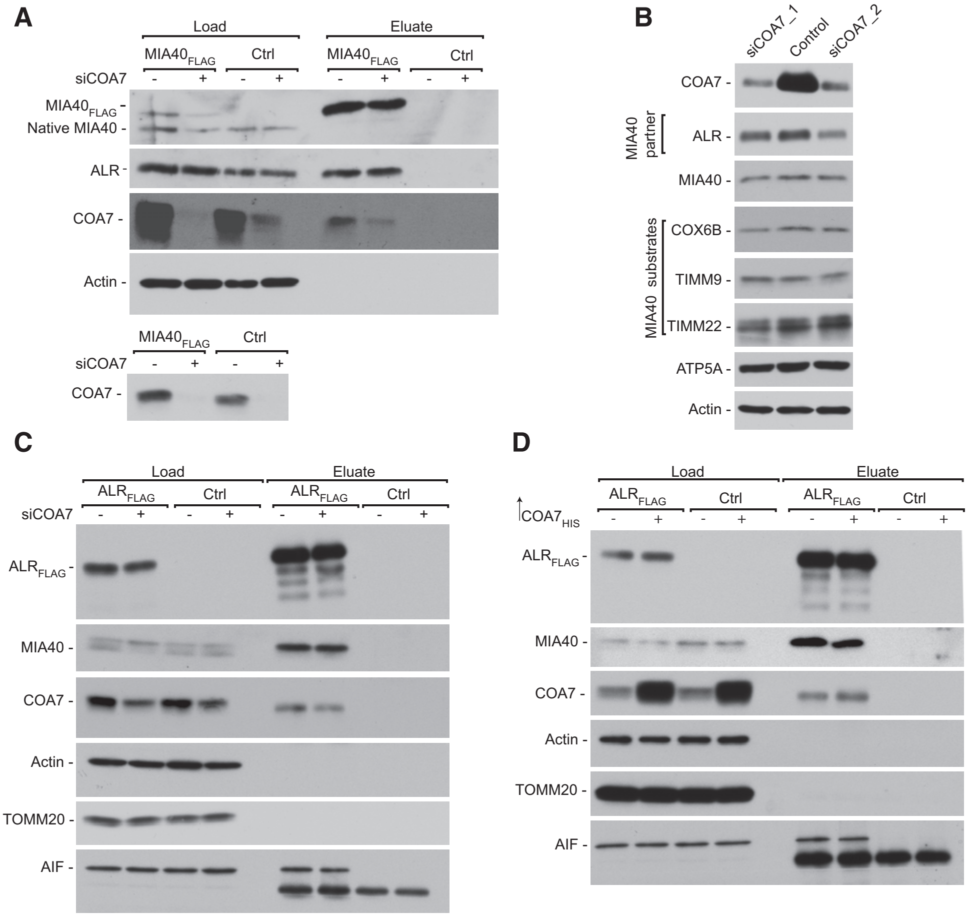

Figure EV2. COA7 does not affect MIA40-ALR interaction.

A Protein extracts were isolated from Flp-In T-REx 293 cells induced to express $MIA40_{FLAG}$ that was transfected with oligonucleotides that targeted COA7 mRNA or control oligonucleotides and subjected to affinity purification. The samples were analyzed by SDS–PAGE and Western blot. Load: 2.5%. Eluate: 100%.

B Cellular protein extracts were isolated from HeLa cells that were transfected with oligonucleotides that targeted different regions of COA7 mRNA or control oligonucleotides. The samples were analyzed by reducing SDS–PAGE and Western blot.

C Protein extracts were isolated from Flp-In T-REx 293 cells induced to express ALR_{FLAG} that was transfected with oligonucleotides that targeted COA7 mRNA or control oligonucleotides and subjected to affinity purification. The samples were analyzed by SDS–PAGE and Western blot. Load: 2.5%. Eluate: 100%.

D Flp-In T-REx 293 cells induced to express ALR_{FLAG} were transfected with a plasmid that encoded $COA7_{HIS}$ or an empty vector. The affinity purification of ALR_{FLAG} was performed, and samples were analyzed by SDS–PAGE and Western blot.

© 2019 The Authors

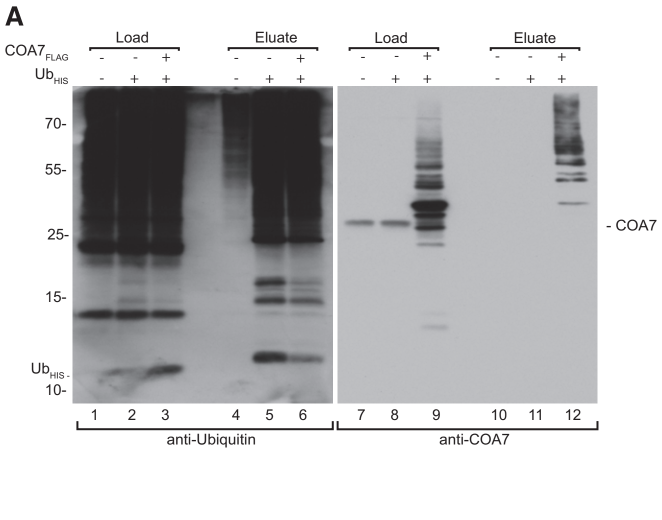

Figure EV3. Involvement of ubiquitin–proteasome system and YME1L in degradation of COA7 and its mutants.

A Cellular protein extracts were isolated from HEK293 cells that expressed $ubiquitin_{HIS}$ (Ub_{His}) and $COA7_{FLAG}$ and subjected to affinity purification. Load: 2.5%. Eluate: 100%.

B Cellular protein extracts were isolated after 48 and 72 h from patient fibroblast that were transfected with oligonucleotides that targeted different regions of YME1L mRNA or control oligonucleotides. The samples were analyzed by reducing SDS–PAGE and Western blot.

 © 2019 The Authors
