# Supplementary material for: Inhibition of proteasome rescues a pathogenic variant of respiratory chain assembly factor COA7
Source: EMBO Mol Med. 2019 Mar 18;11(5):e9561. doi: 10.15252/emmm.201809561 (PMC6505684; doi:10.15252/emmm.201809561)
Supplement: Supplementary file 5 — Dataset EV3 [file EMMM-11-e9561-s005.docx]

Mohanraj et al. 2019

Dataset EV3

MIA40 deletion mutant allele sequence analysis.

Sequence alignment between wild type MIA40 (WT_WT) and MIA40 mutant allele PCR product (WT_Del)


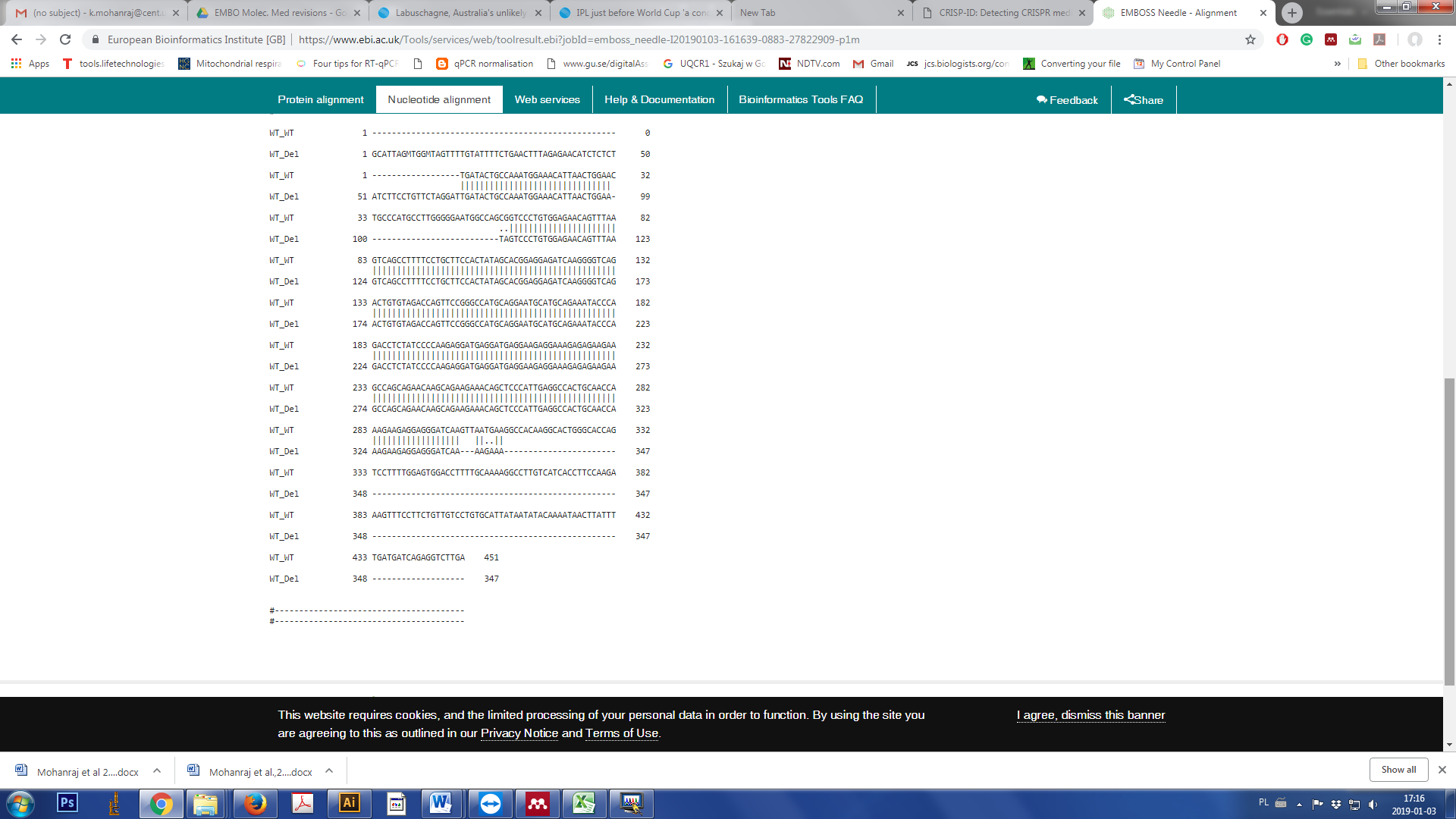


Deleted fragment in one allele of HEK MIA40 WT/Del^53-60^ cells: CTG CCC ATG CCT TGG GGG AAT GGC CAG

Two base substitution in one allele of HEK MIA40 WT/Del^53-60^ cells: CG TA
