# Supplementary material for: Inhibition of proteasome rescues a pathogenic variant of respiratory chain assembly factor COA7
Source: EMBO Mol Med. 2019 Mar 18;11(5):e9561. doi: 10.15252/emmm.201809561 (PMC6505684; doi:10.15252/emmm.201809561)
Supplement: Supplementary file 7 — Source Data for Figure 1 [file EMMM-11-e9561-s006.pdf]

Corresponding to Figure 1B

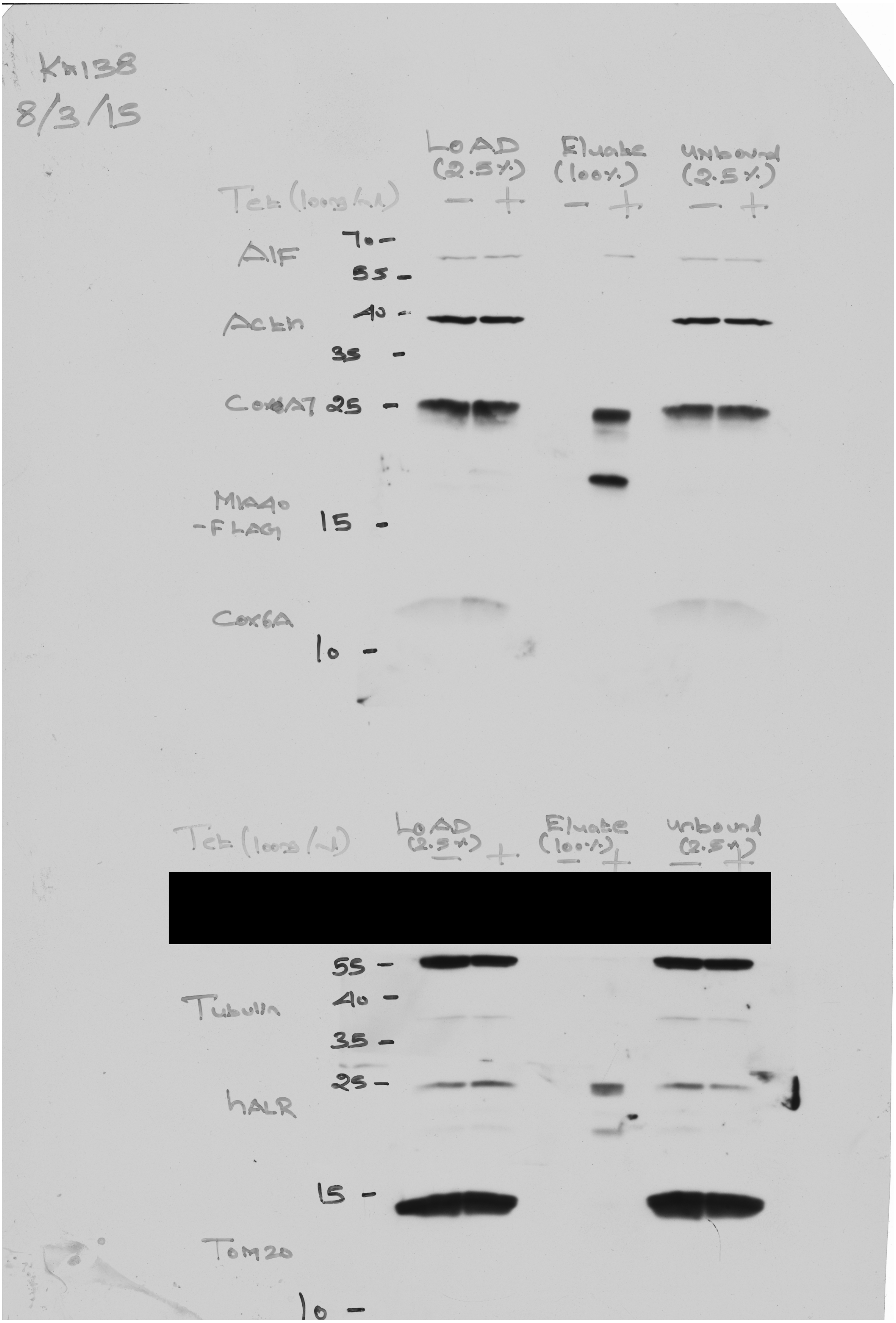

Corresponding to Figure 1B

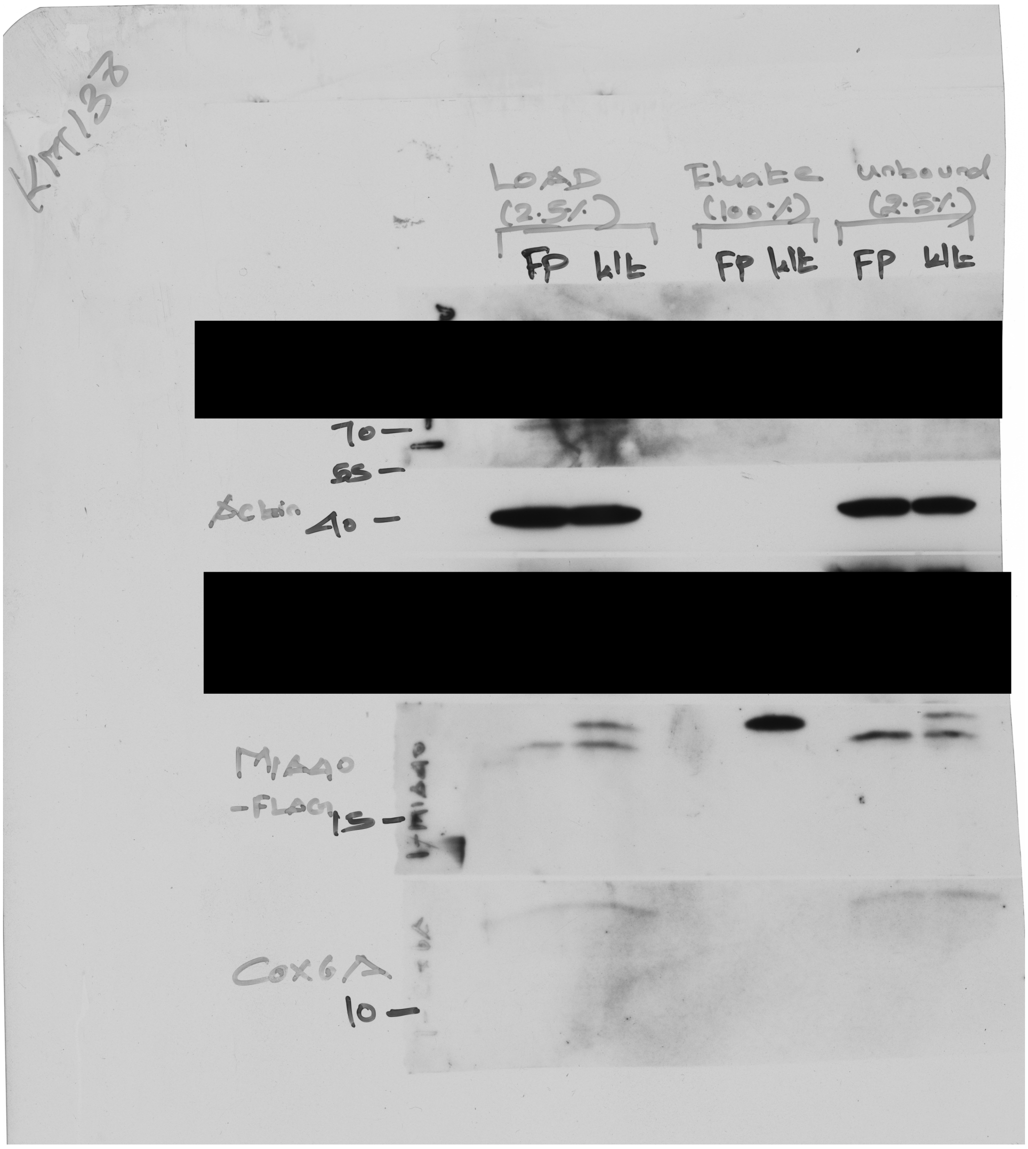

Corresponding to Figure 1C

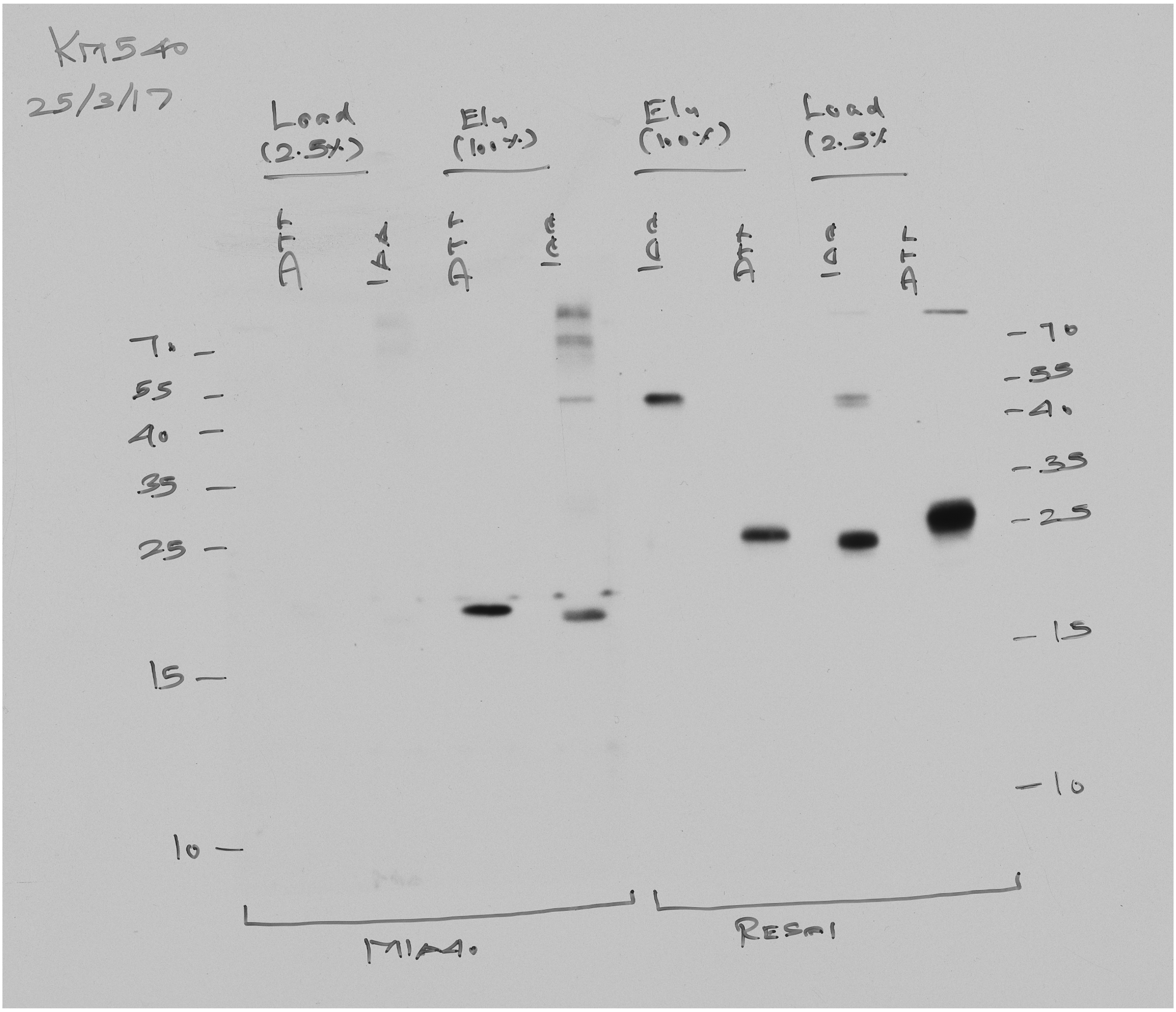

RESA1 is an alternative name for COA7

Corresponding to Figure 1D

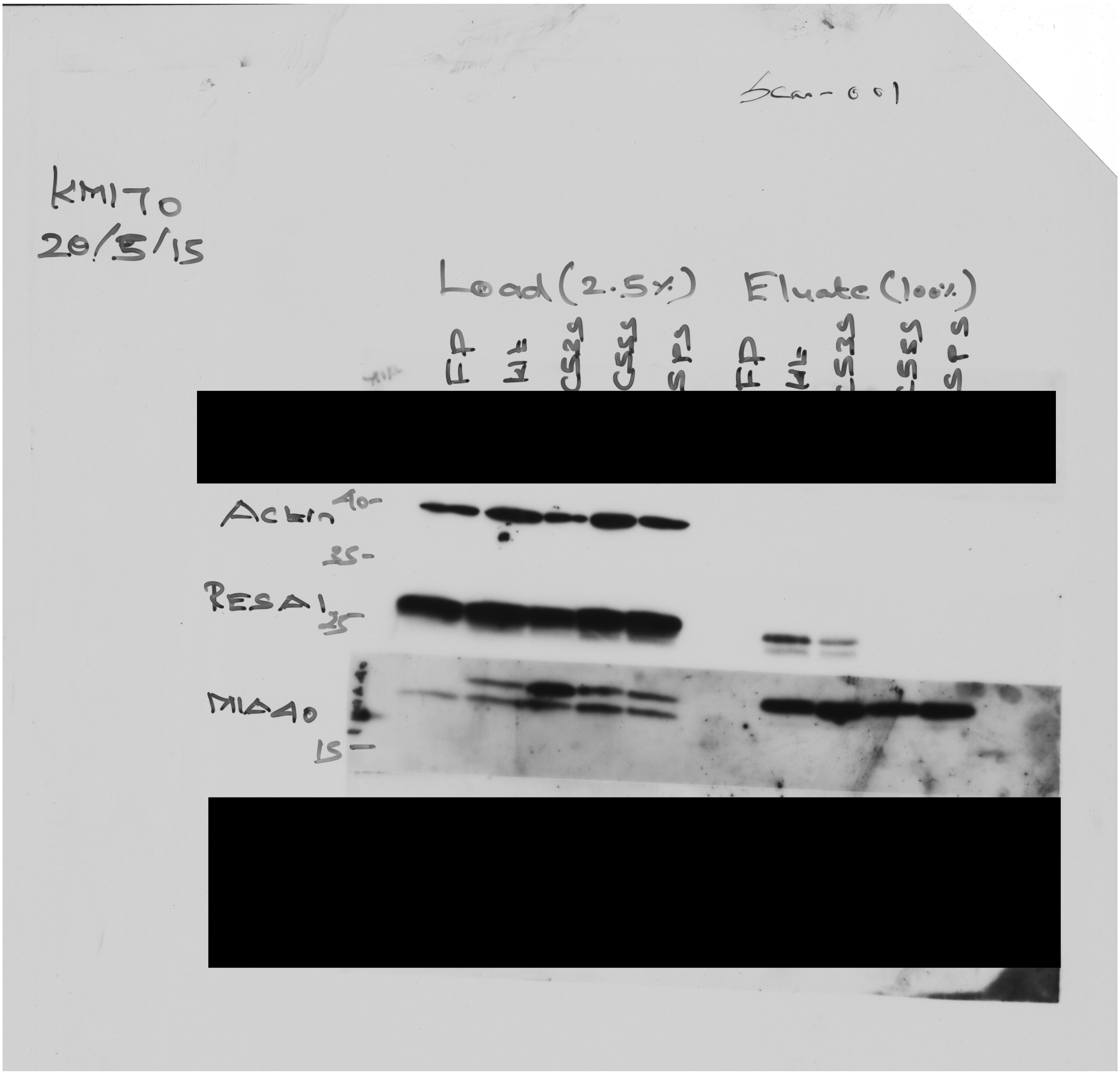

Corresponding to Figure 1D

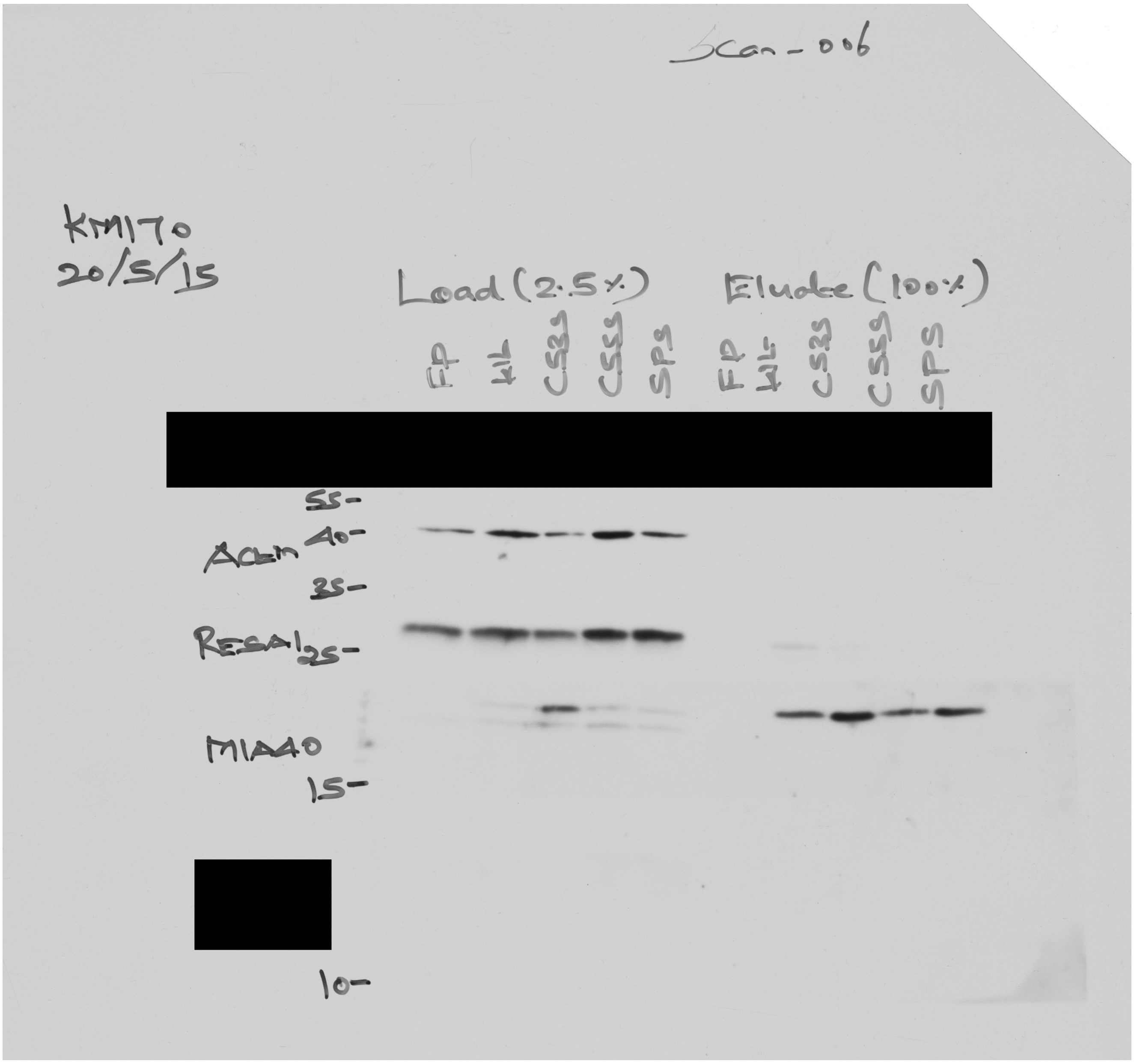

RESA1 is an alternative name for COA7
