# Supplementary material for: Inhibition of proteasome rescues a pathogenic variant of respiratory chain assembly factor COA7
Source: EMBO Mol Med. 2019 Mar 18;11(5):e9561. doi: 10.15252/emmm.201809561 (PMC6505684; doi:10.15252/emmm.201809561)
Supplement: Supplementary file 8 — Source Data for Figure 2 [file EMMM-11-e9561-s007.pdf]

Corresponding to Figure 2C

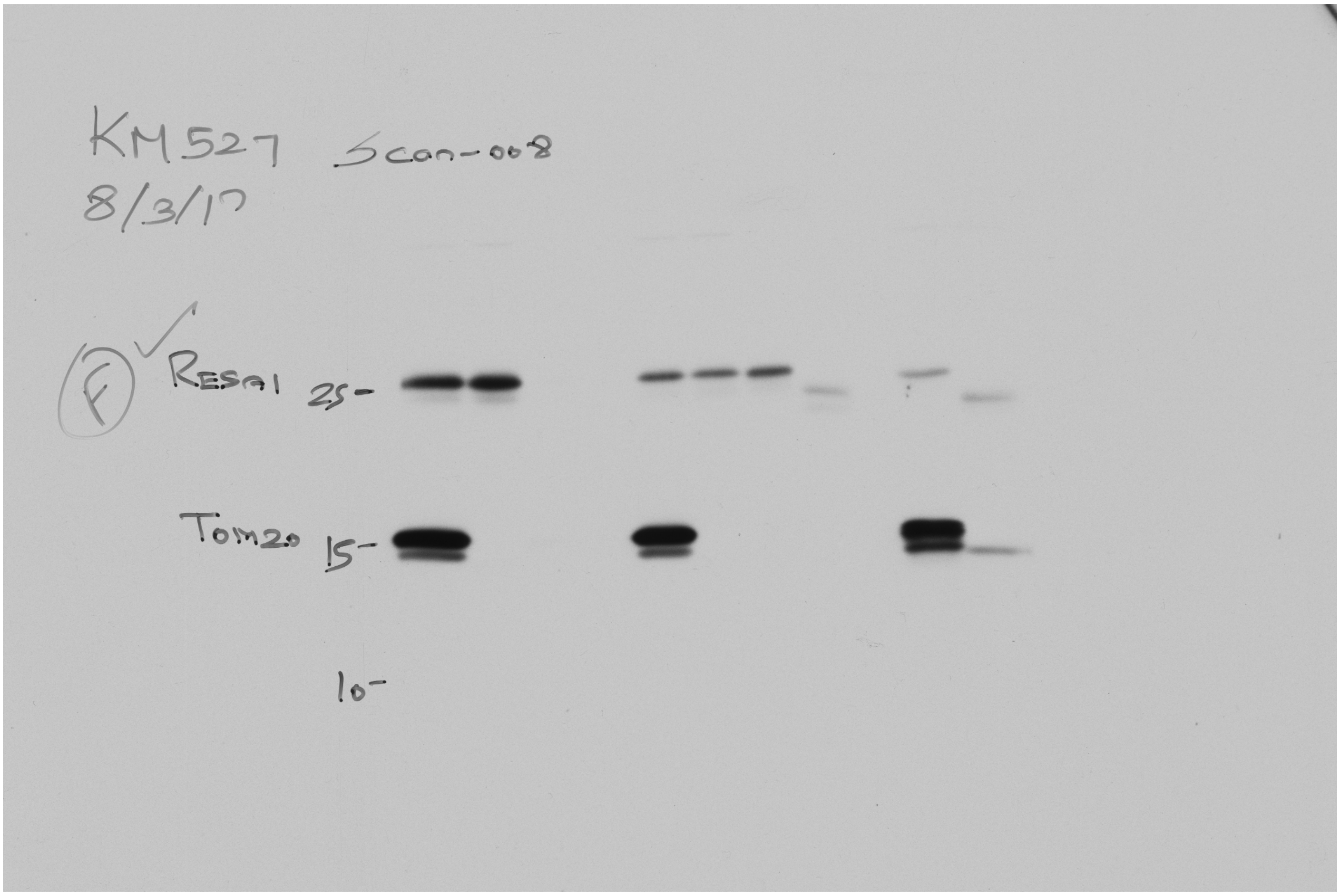

RESA1 is an alternative name for COA7

Corresponding to Figure 2C

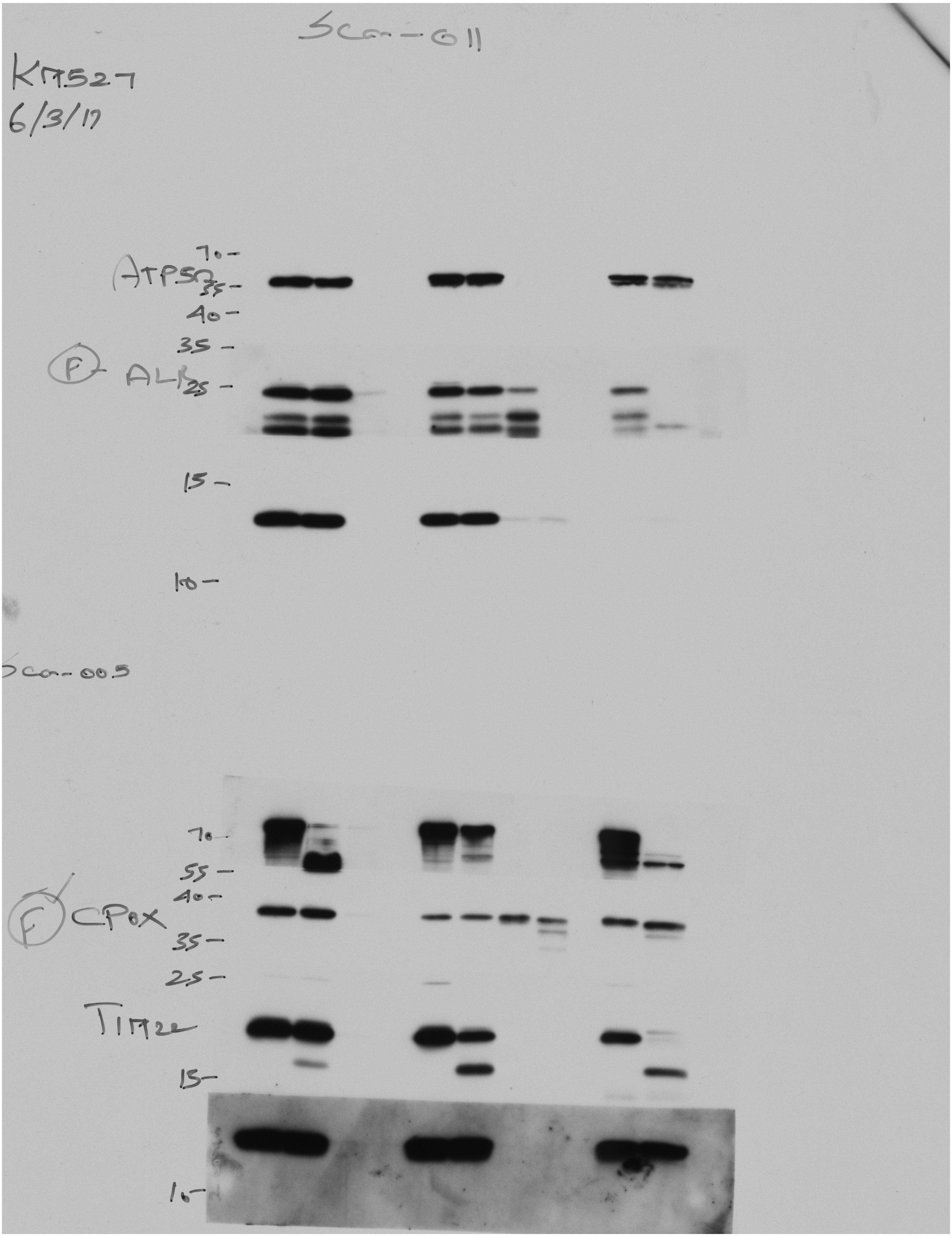

Corresponding to Figure 2D

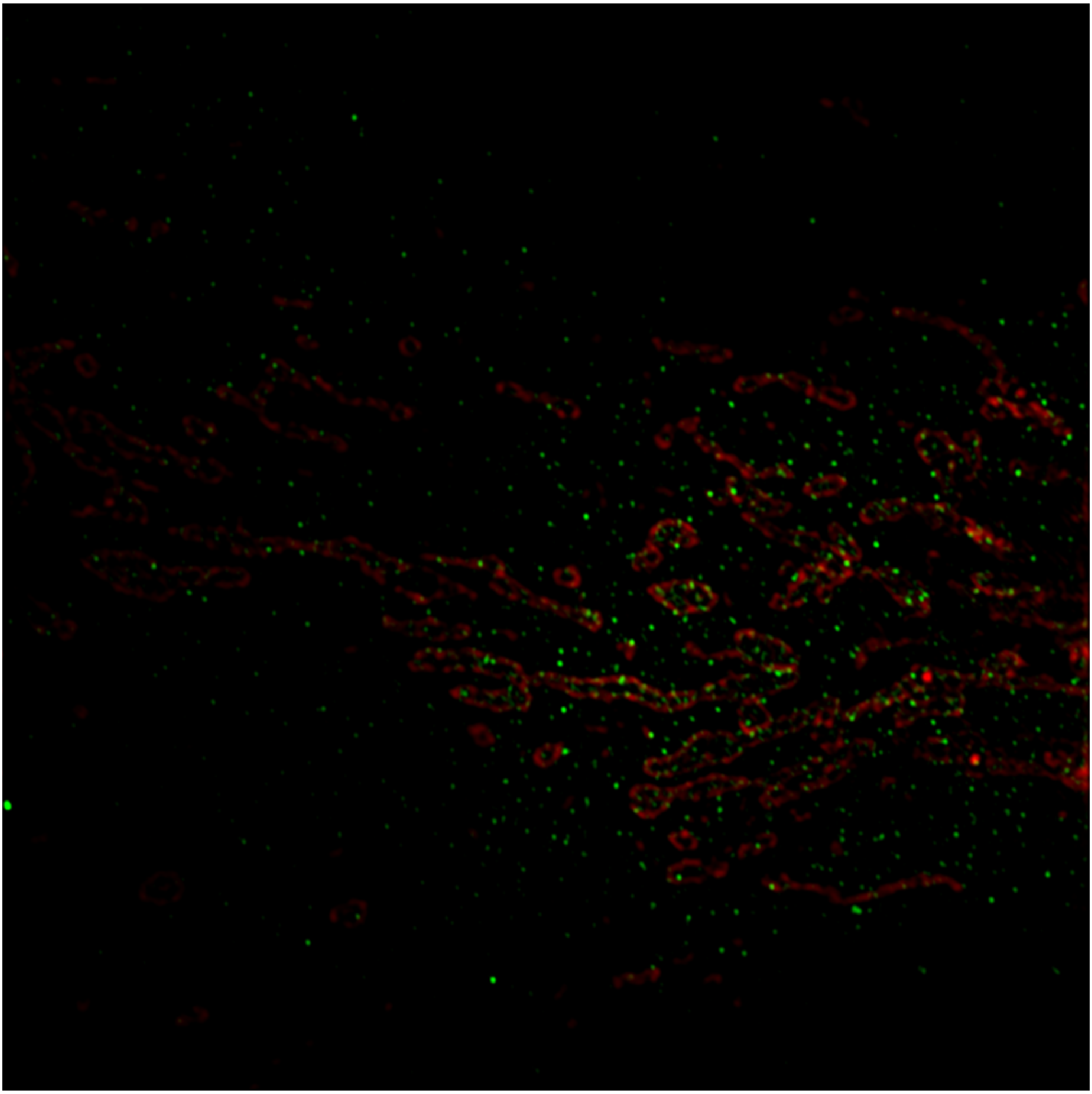

COA7 (green) x Cox8a (red)

Corresponding to Figure 2D

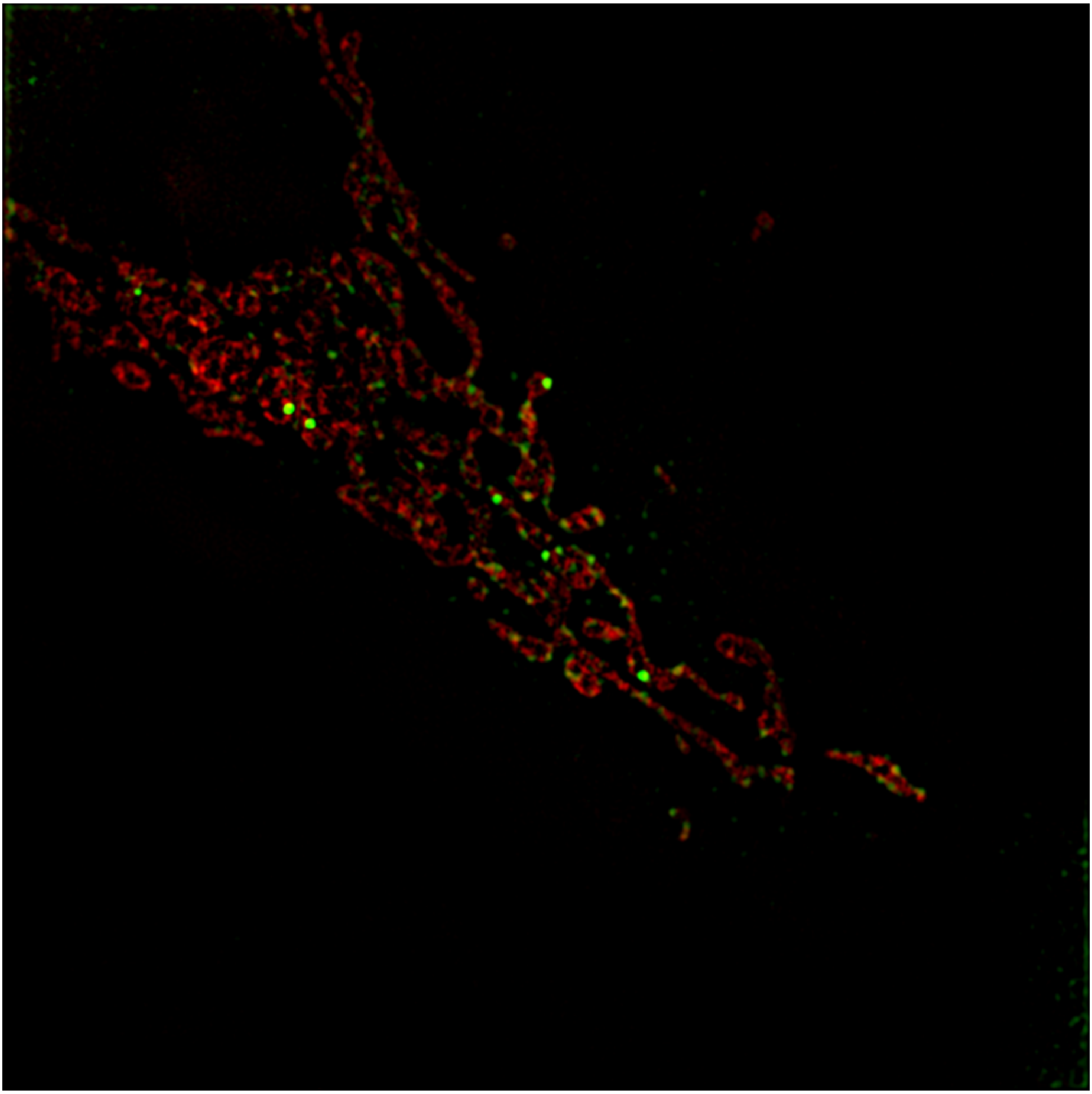

COA7 (green) x Matrix (red)

Corresponding to Figure 2D

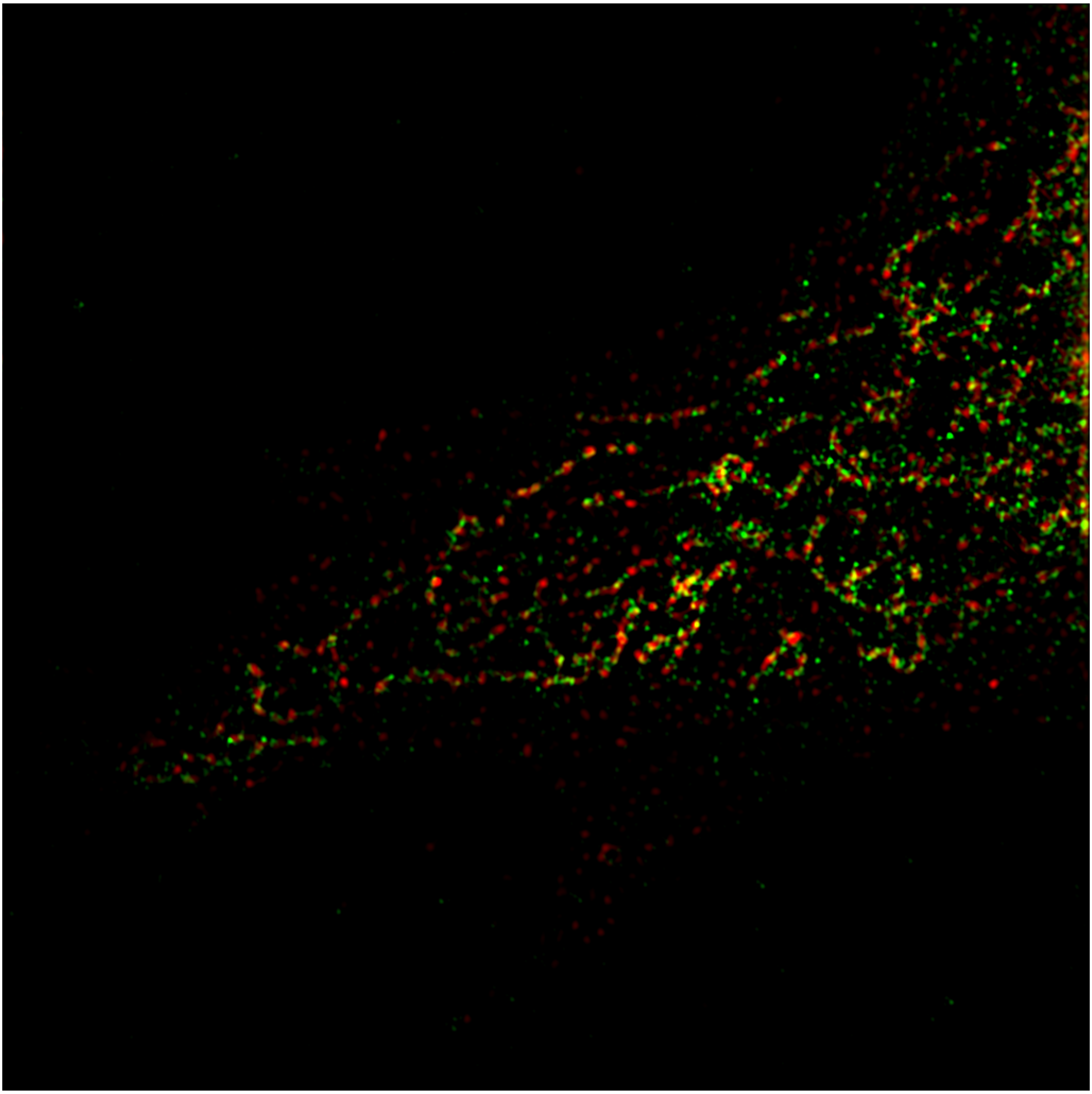

COA7 (green) x SMAC (red)

Corresponding to Figure 2D

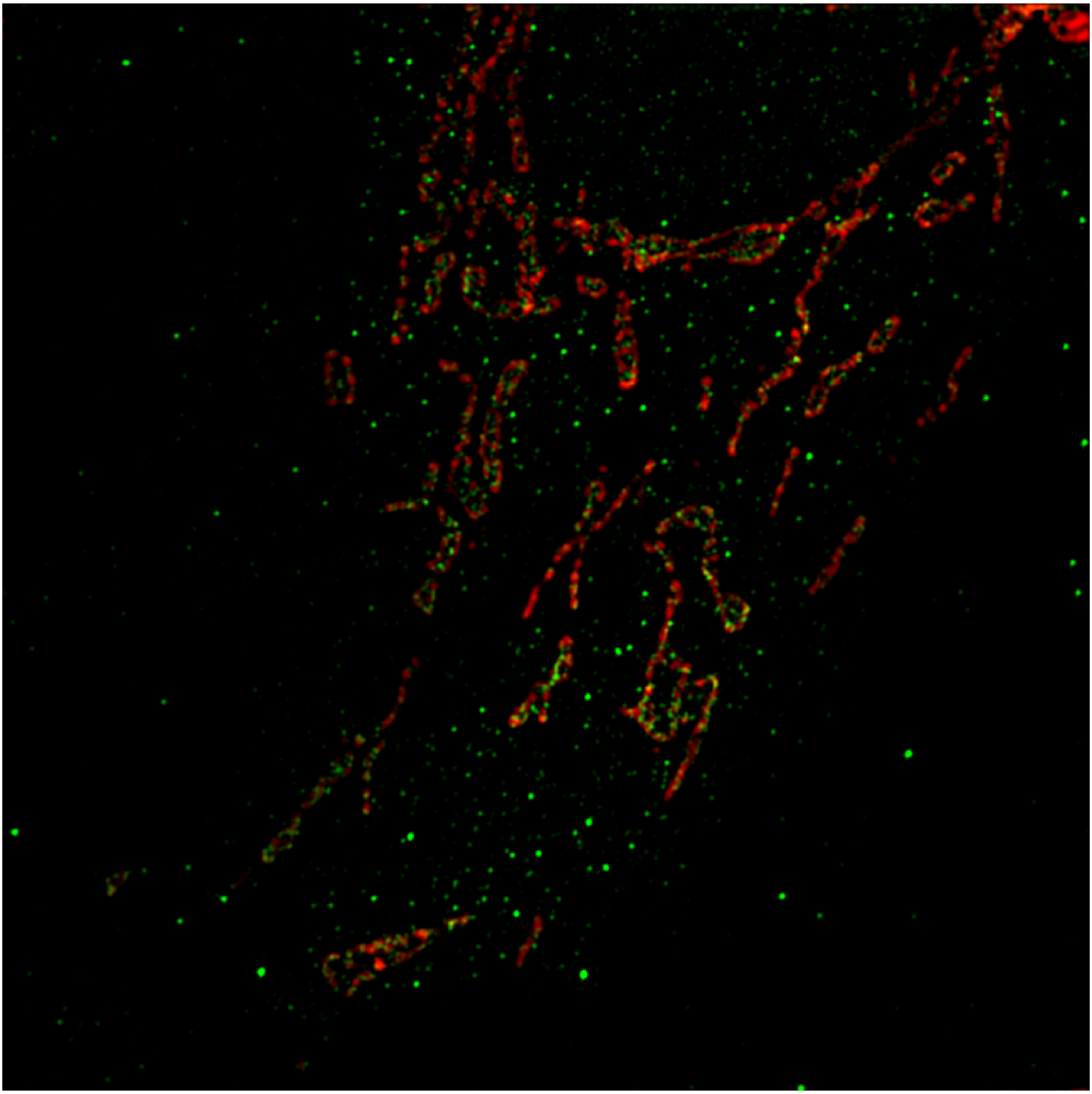

COA7 (green) x TOMM20 (red)

Corresponding to Figure 2D

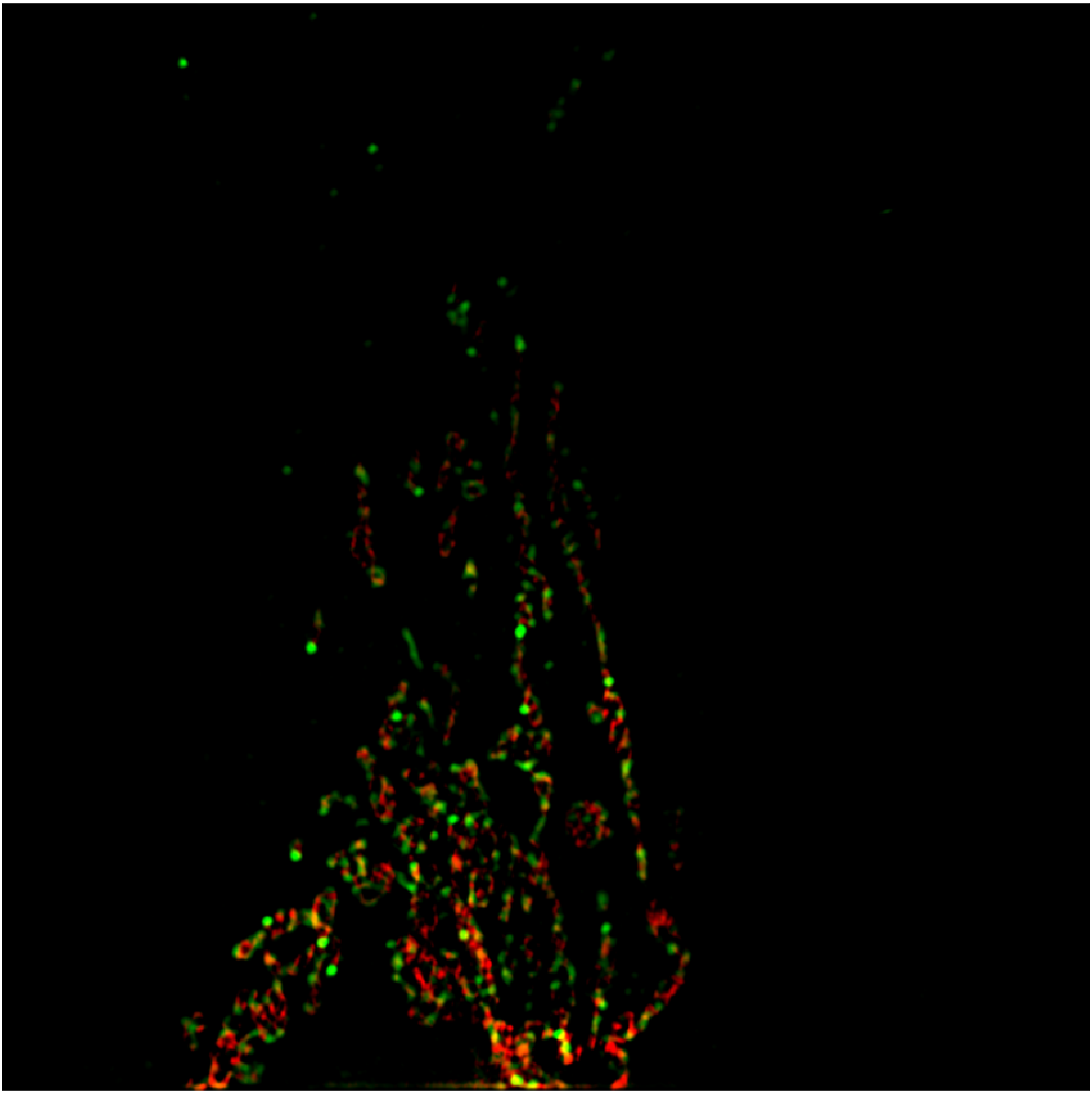

COA7HA (green) x Matrix (red)

Corresponding to Figure 2D

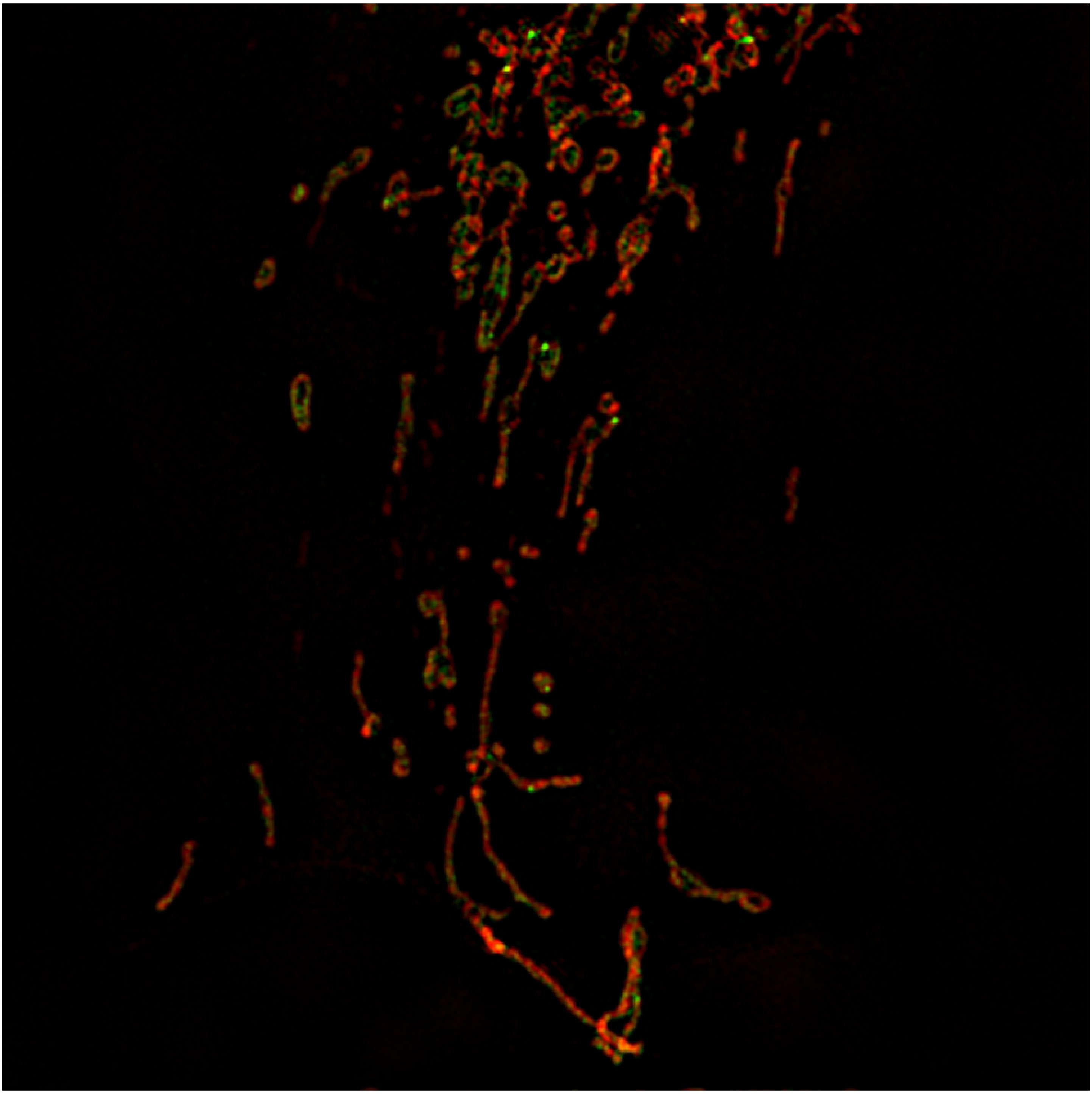

COA7HA (green) x TOMM20 (red)

Corresponding to Figure 2D

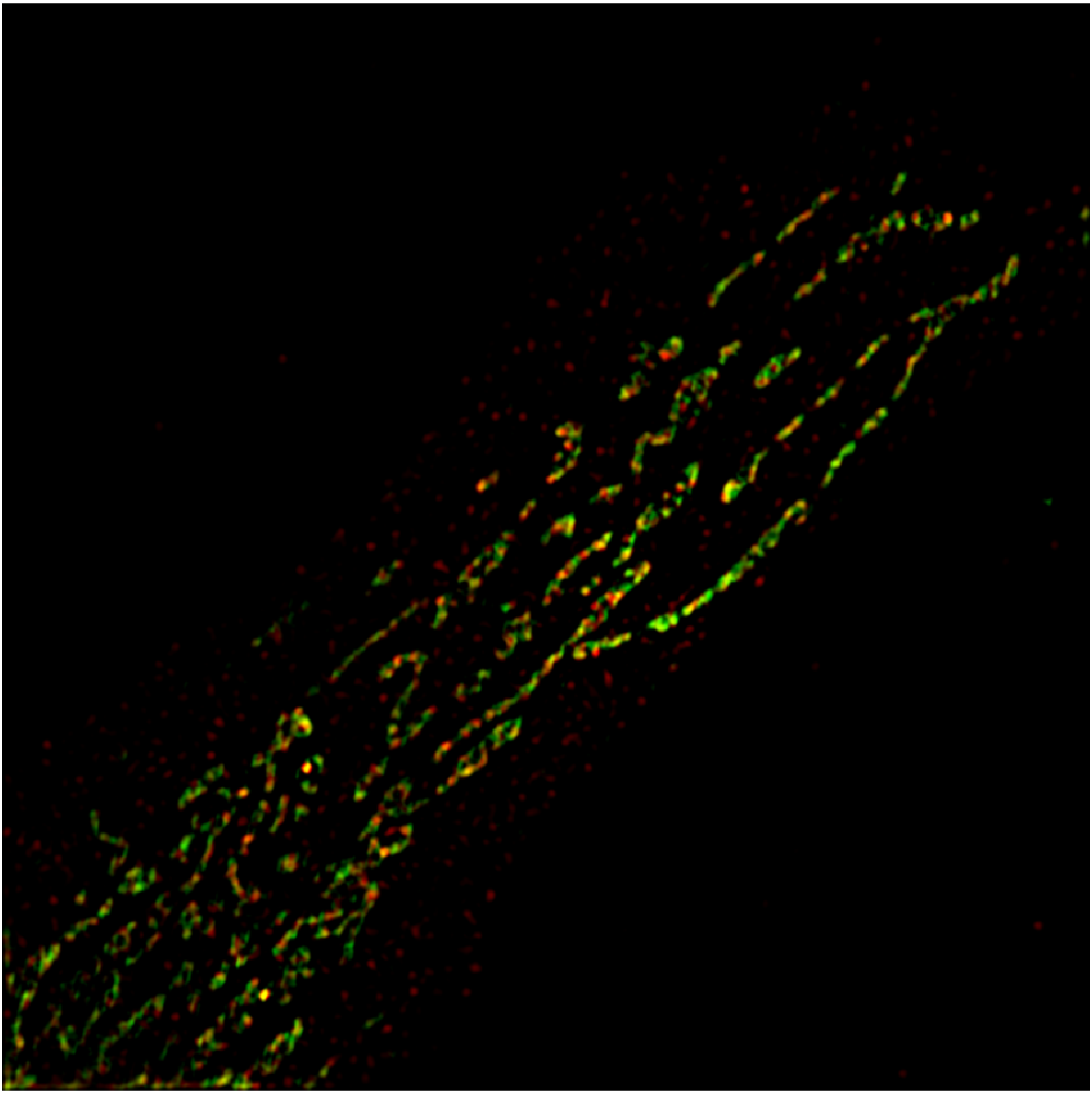

COA7HA (gren) x SMAC (red)

Corresponding to Figure 2D

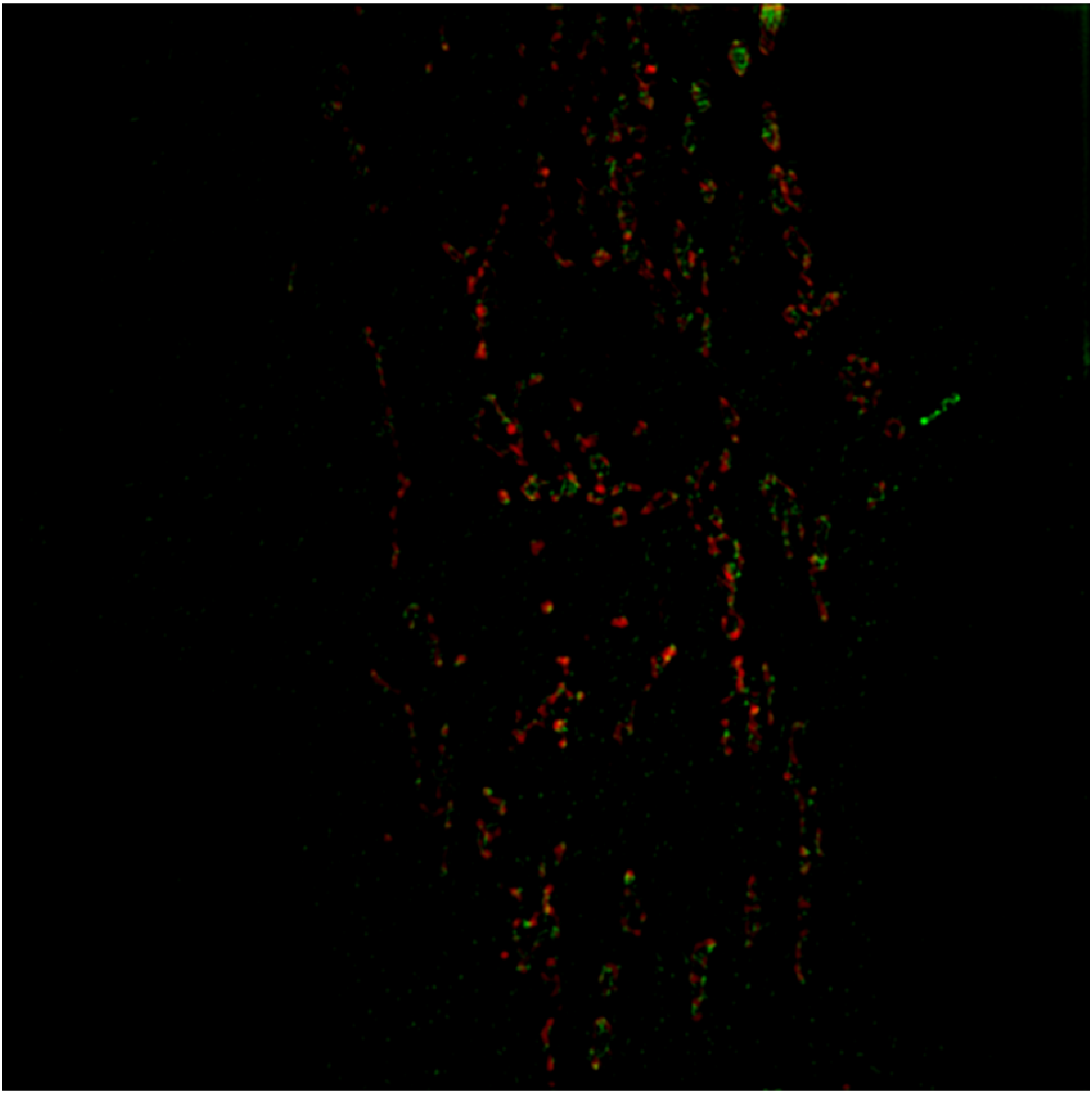

COA7HA (green) xCox8a (red)
