# Supplementary material for: Inhibition of proteasome rescues a pathogenic variant of respiratory chain assembly factor COA7
Source: EMBO Mol Med. 2019 Mar 18;11(5):e9561. doi: 10.15252/emmm.201809561 (PMC6505684; doi:10.15252/emmm.201809561)

Corresponding to Figure 3C

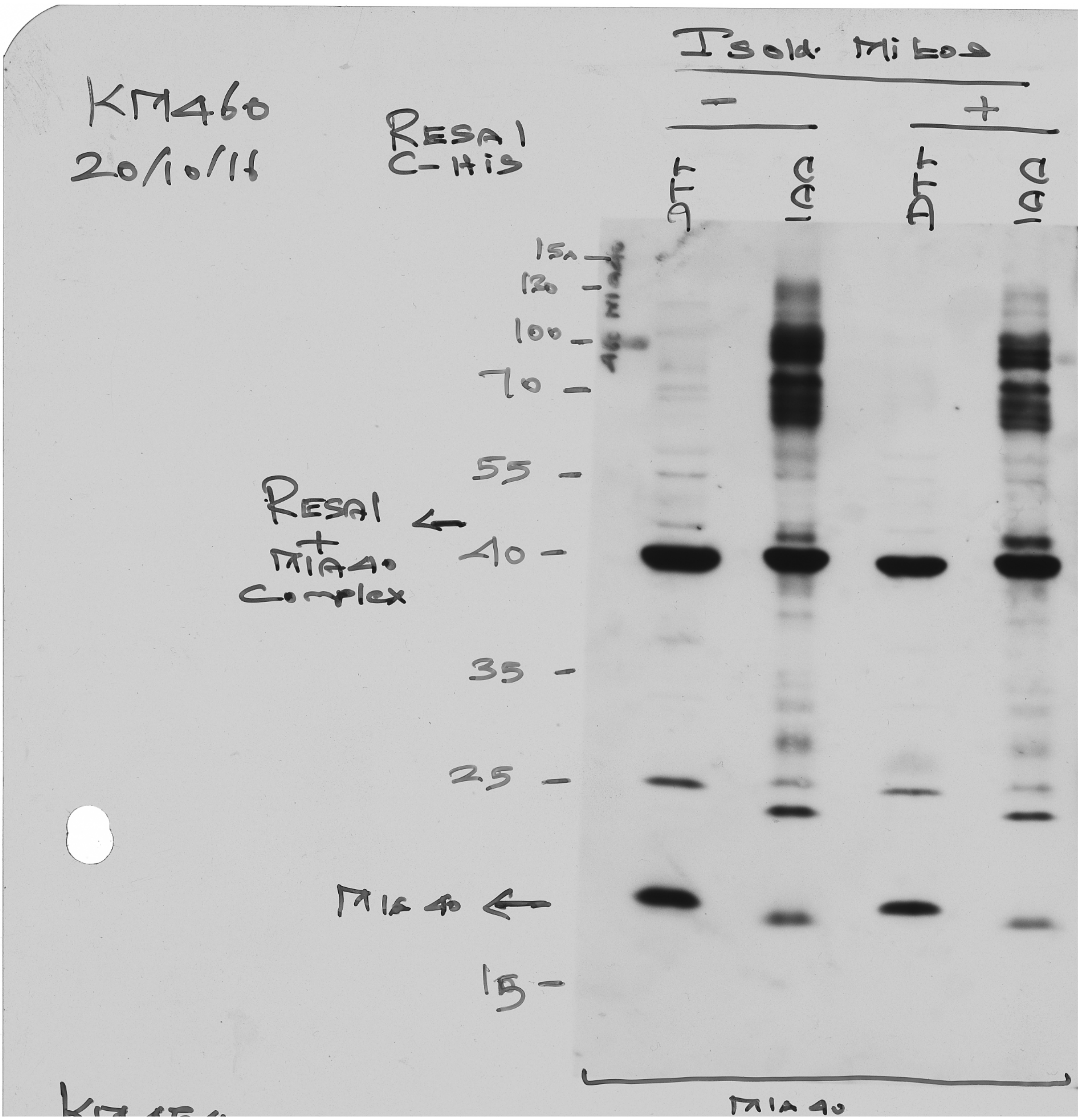

RESA1 is an alternative name for COA7

Corresponding to Figure 3D

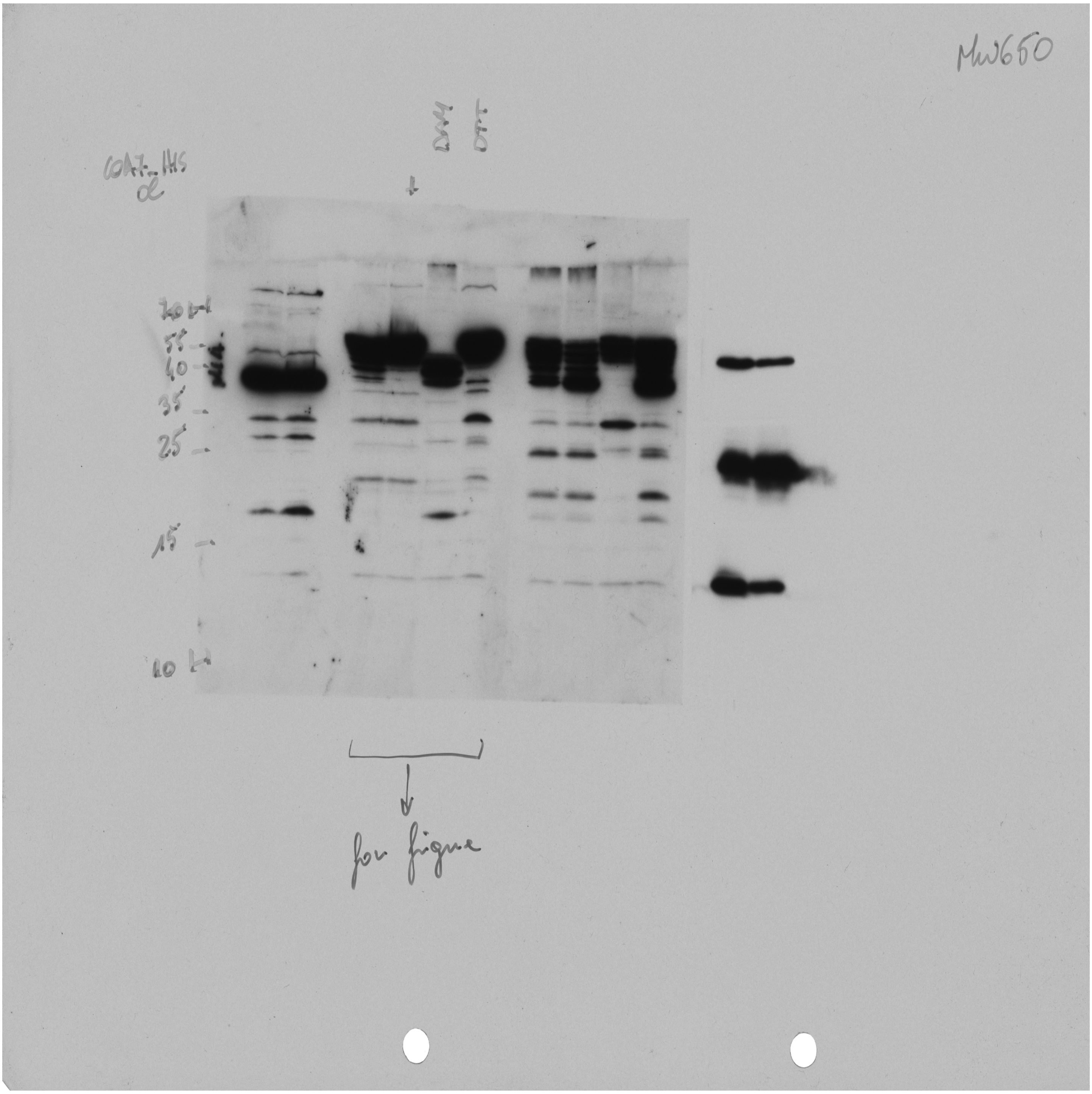

Corresponding to Figure 3E

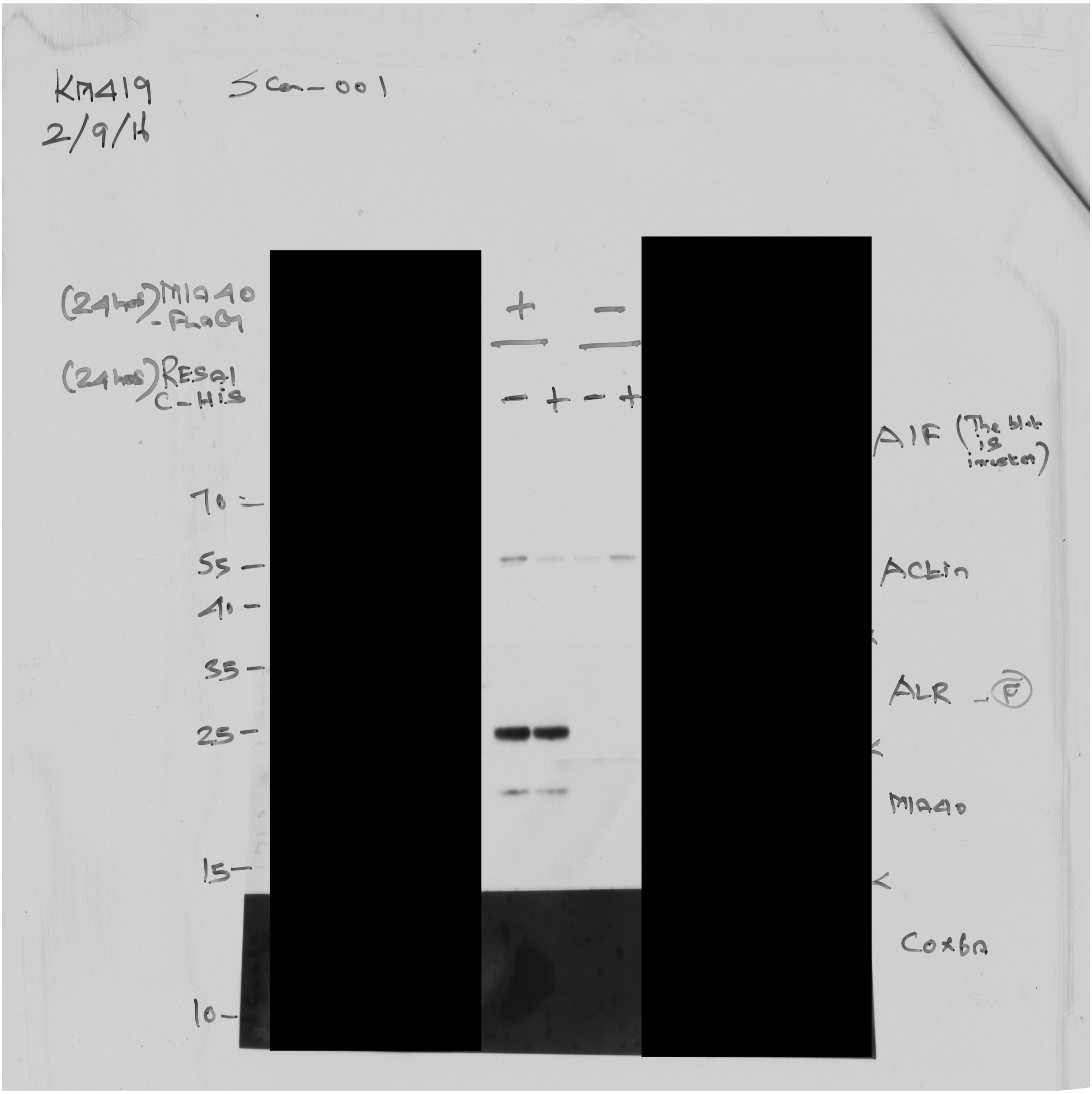

RESA1 is an alternative name for COA7

Corresponding to Figure 3E

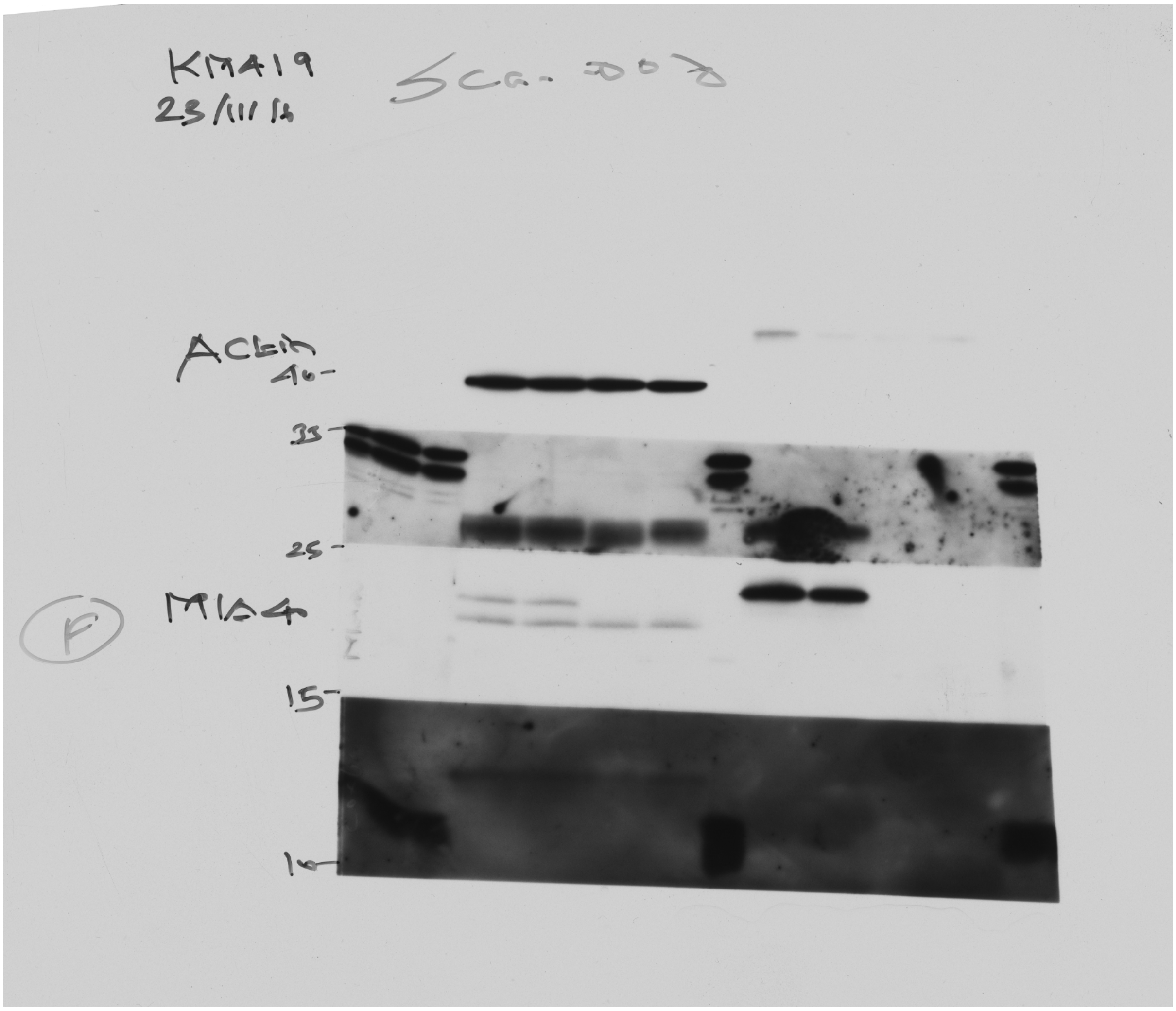

Supplement: Supplementary file 9 — Source Data for Figure 3 [file EMMM-11-e9561-s008.pdf]
