# Supplementary material for: Inhibition of proteasome rescues a pathogenic variant of respiratory chain assembly factor COA7
Source: EMBO Mol Med. 2019 Mar 18;11(5):e9561. doi: 10.15252/emmm.201809561 (PMC6505684; doi:10.15252/emmm.201809561)

Corresponding to Figure 5E

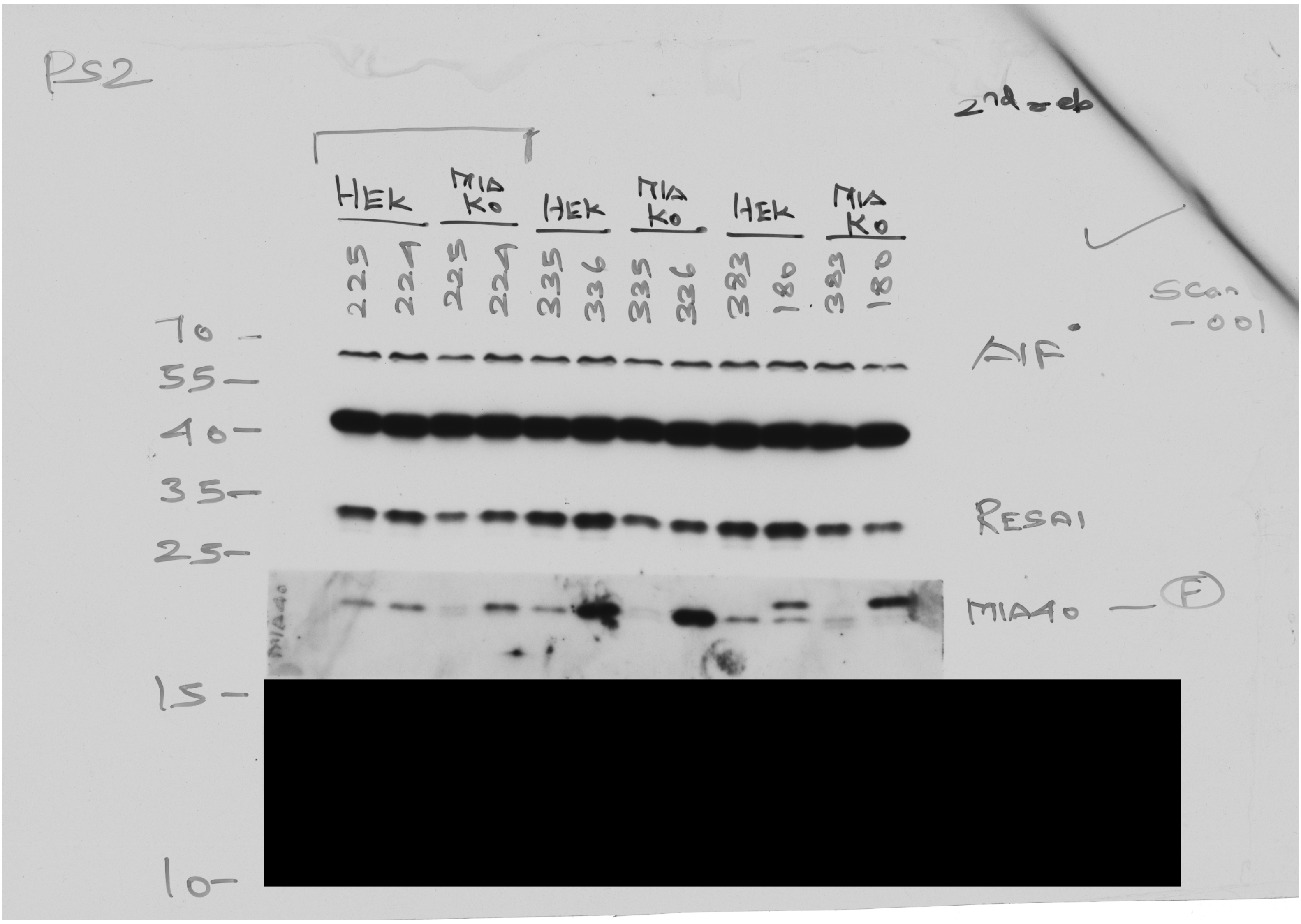

RESA1 is an alternative name for COA7

Corresponding to Figure 5E

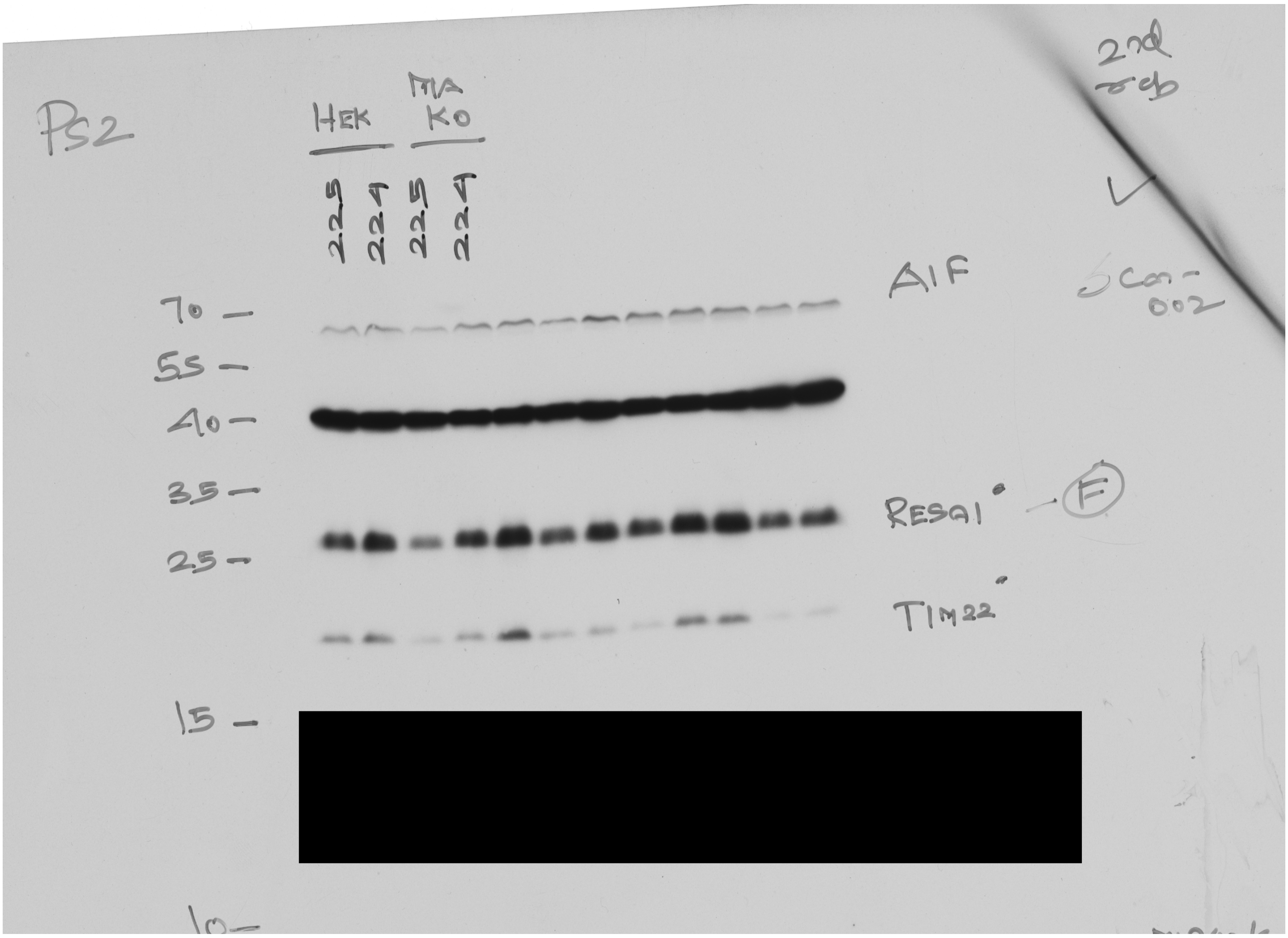

RESA1 is an alternative name for COA7

Corresponding to Figure 5E

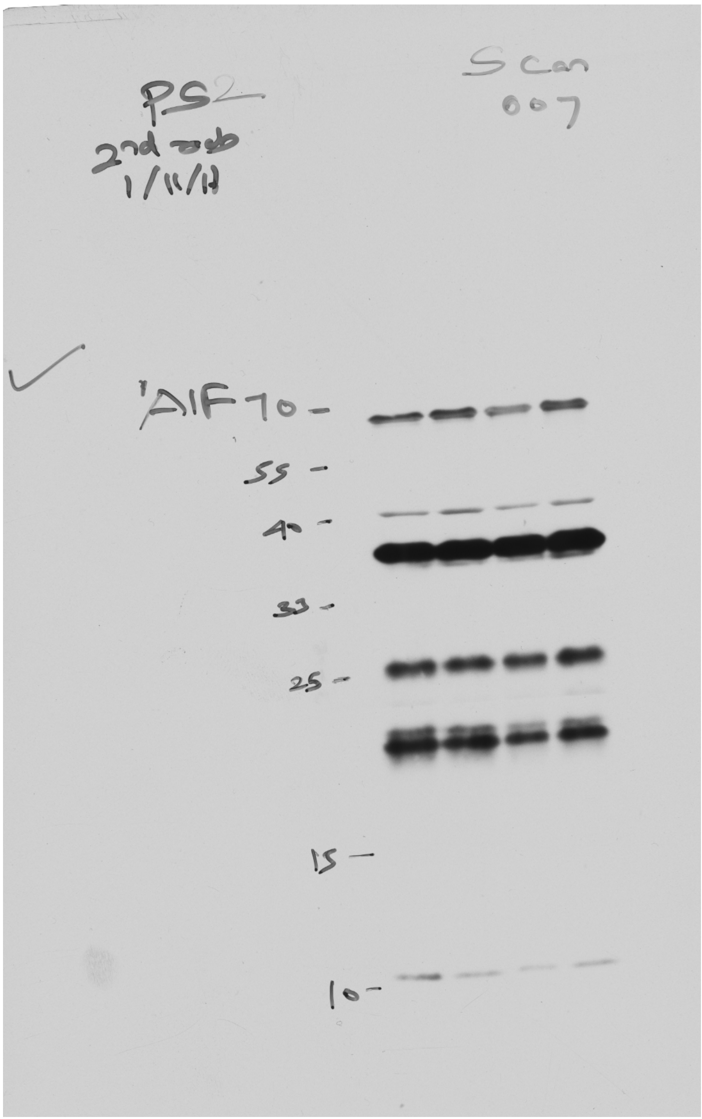

Supplement: Supplementary file 11 — Source Data for Figure 5 [file EMMM-11-e9561-s010.pdf]
