# Supplementary material for: Inhibition of proteasome rescues a pathogenic variant of respiratory chain assembly factor COA7
Source: EMBO Mol Med. 2019 Mar 18;11(5):e9561. doi: 10.15252/emmm.201809561 (PMC6505684; doi:10.15252/emmm.201809561)

Corresponding to Figure 6C

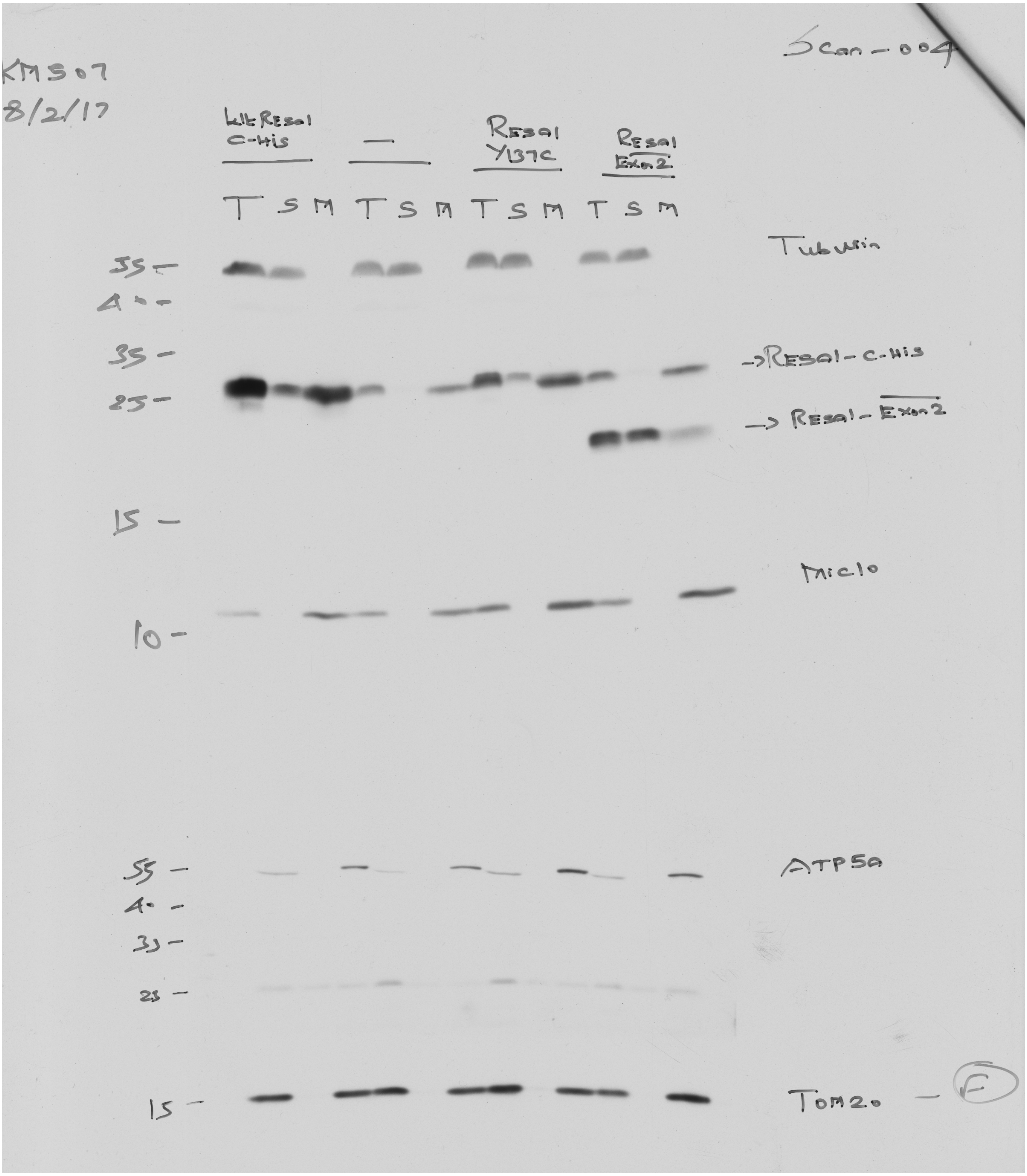

RESA1 is an alternative name for COA7

Corresponding to Figure 6C

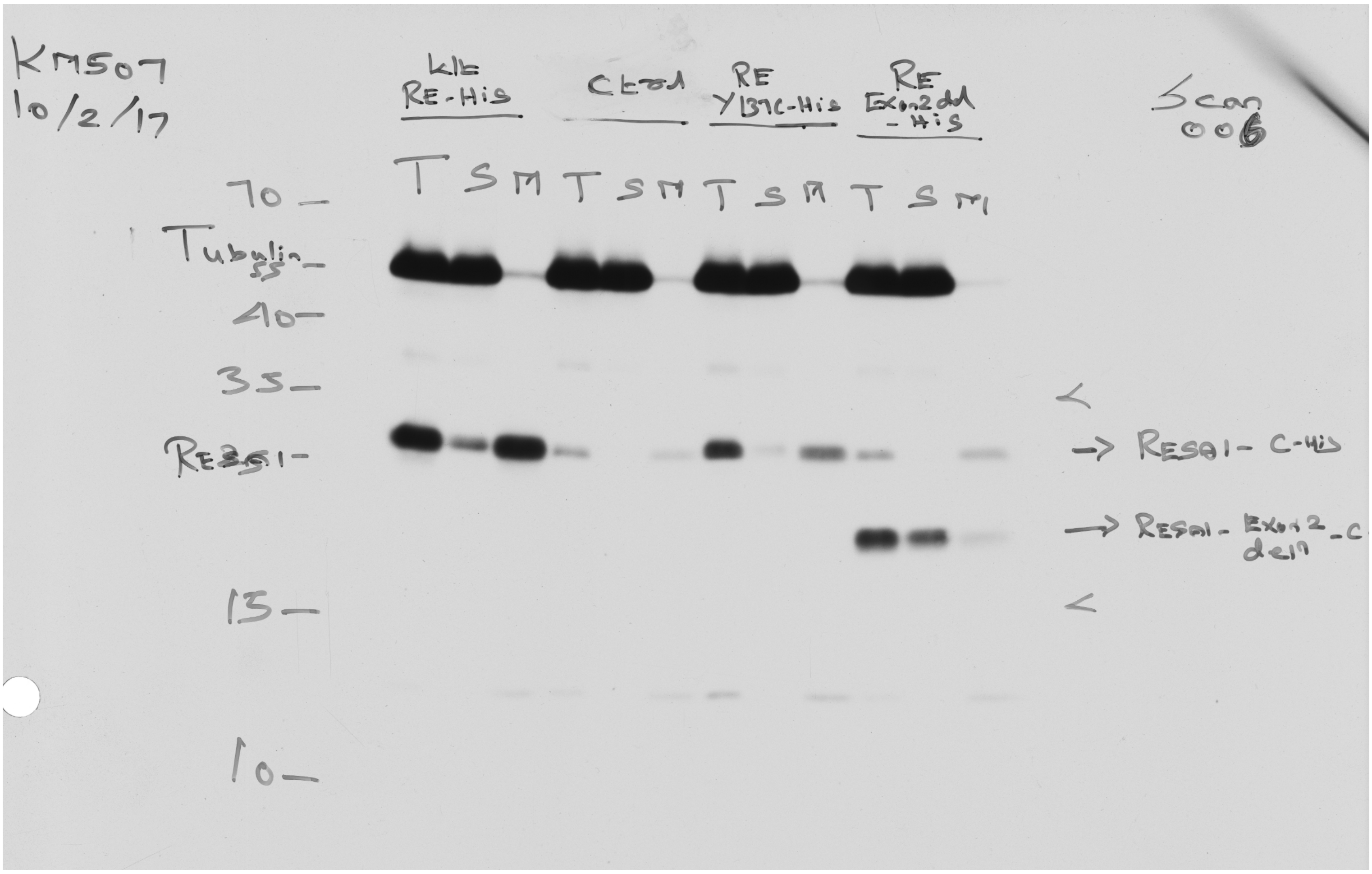

RESA1 is an alternative name for COA7

Corresponding to Figure 6C

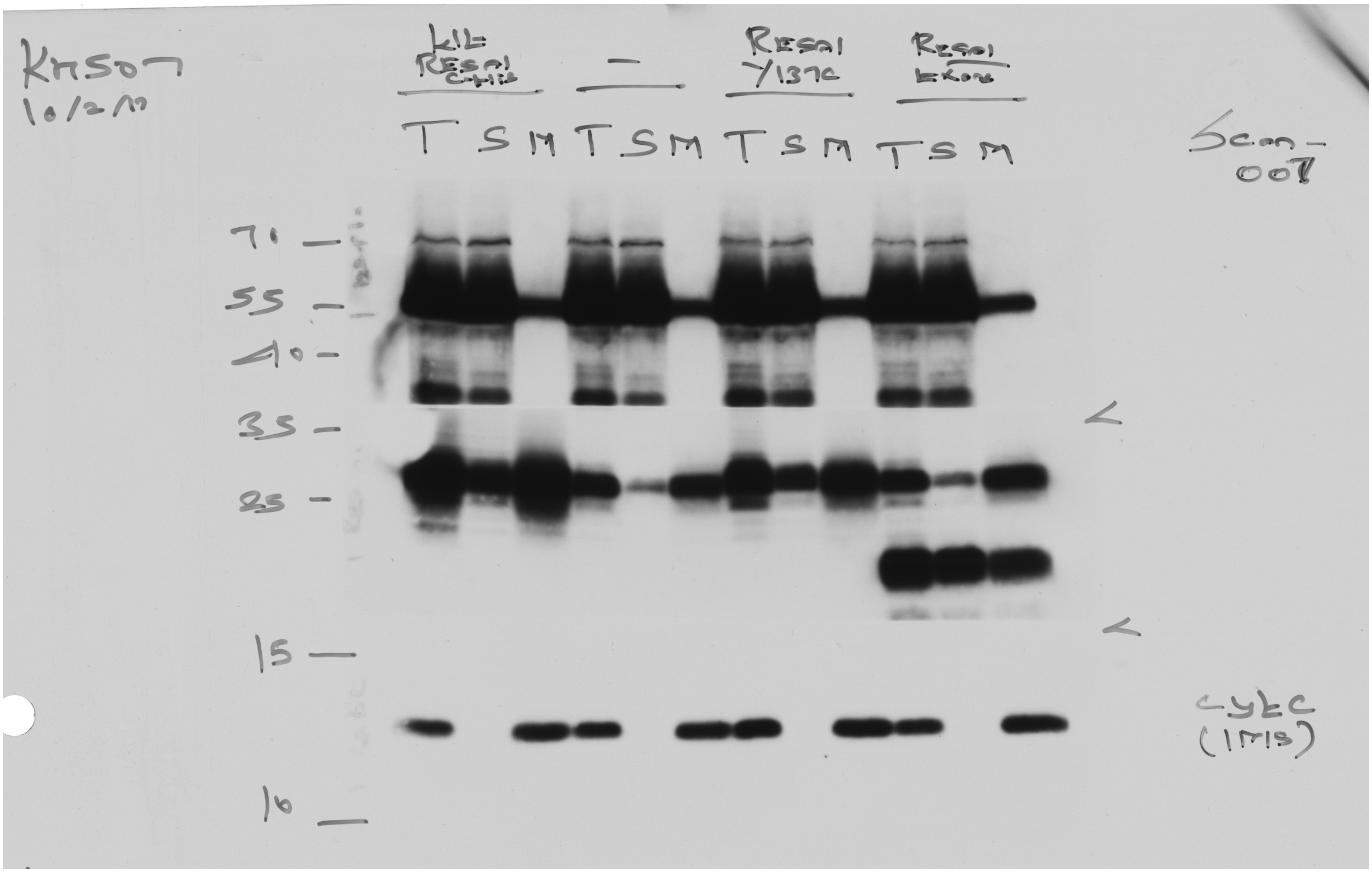

Corresponding to Figure 6E

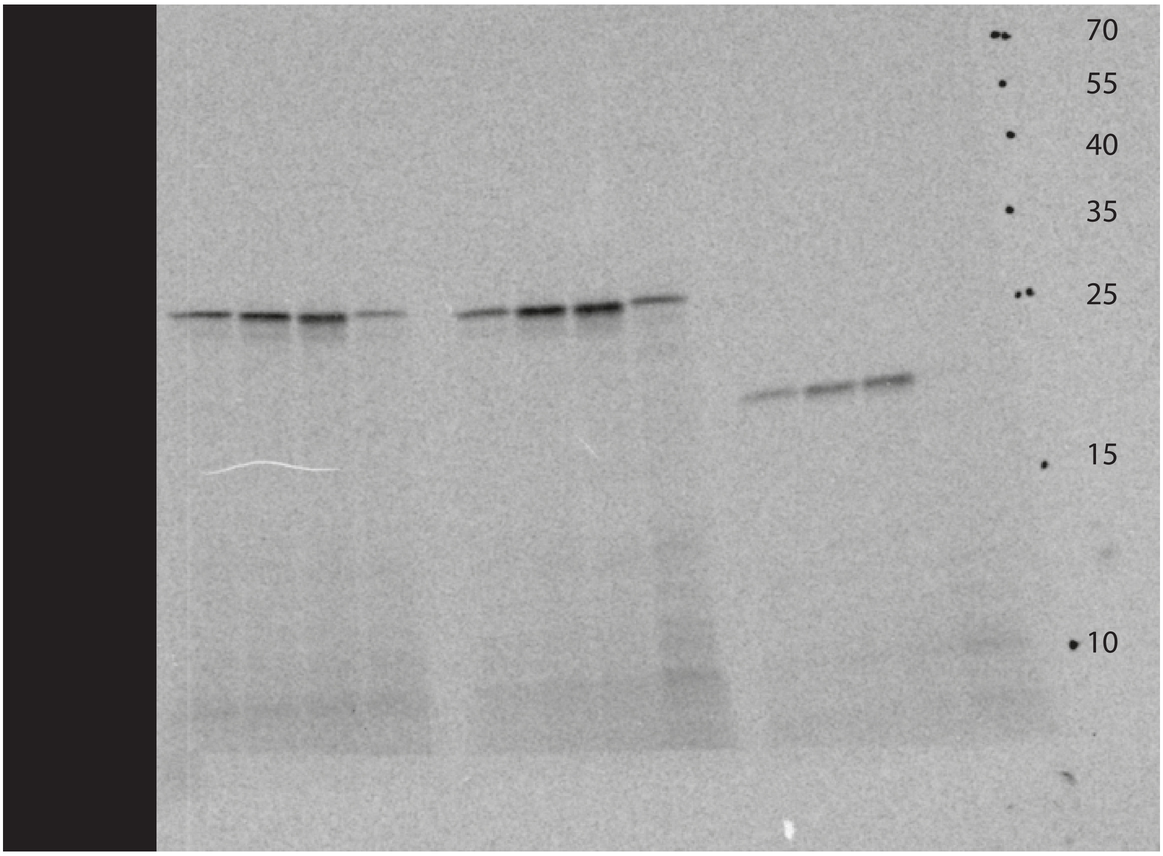

Supplement: Supplementary file 12 — Source Data for Figure 6 [file EMMM-11-e9561-s011.pdf]
